# Super-emitters in natural gas infrastructure are caused by abnormal process conditions

**DOI:** 10.1038/ncomms14012

**Published:** 2017-01-16

**Authors:** Daniel Zavala-Araiza, Ramón A Alvarez, David R. Lyon, David T. Allen, Anthony J. Marchese, Daniel J. Zimmerle, Steven P. Hamburg

**Affiliations:** 1 Environmental Defense Fund, 301 Congress Avenue, Suite 1300, Austin, Texas 78701, USA; 2 Center for Energy and Environmental Resources, The University of Texas at Austin, 10100 Burnet Road, Austin, Texas 78758, USA; 3 Department of Mechanical Engineering, Colorado State University, Fort Collins, Colorado 80523, USA; 4 The Energy Institute, Colorado State University, Fort Collins, Colorado 80523, USA

## Abstract

Effectively mitigating methane emissions from the natural gas supply chain requires addressing the disproportionate influence of high-emitting sources. Here we use a Monte Carlo simulation to aggregate methane emissions from all components on natural gas production sites in the Barnett Shale production region (Texas). Our total emission estimates are two-thirds of those derived from independent site-based measurements. Although some high-emitting operations occur by design (condensate flashing and liquid unloadings), they occur more than an order of magnitude less frequently than required to explain the reported frequency at which high site-based emissions are observed. We conclude that the occurrence of abnormal process conditions (for example, malfunctions upstream of the point of emissions; equipment issues) cause additional emissions that explain the gap between component-based and site-based emissions. Such abnormal conditions can cause a substantial proportion of a site's gas production to be emitted to the atmosphere and are the defining attribute of super-emitting sites.

## Abstract (web summary)

A large proportion of methane emissions from natural gas production sites are released by a fraction of high-emitting sources. Here, using Monte Carlo simulations, the authors reveal that super-emitters occur due to abnormal process conditions, explaining component and site-based inventory discrepancies.

Quantifying methane (CH_4_) emissions from the natural gas supply chain is an active area of research^1^,^2^,^3^,^4^,^5^,^6^,^7^,^8^,^9^,^10^,^11^,^12^,^13^,^14^,^15^,^16^,^17^,^18^,^19^,^20^,^21^, with consistent findings that high-emitting sources disproportionately affect overall emissions. As policy-relevant methane emission estimates are almost exclusively based on component-level inventories^22^,^23^, understanding the component-based behaviours that in aggregate comprise site-level emissions is necessary to guide effective emission mitigation policies. Especially important is determining which components are responsible for the bulk of methane emissions from the highest emitting sites.

In the Barnett Shale (Texas, USA), a recent study of CH_4_ emissions from natural gas infrastructure found that emission estimates derived primarily from facility/site-based measurements agreed within statistical uncertainty with basin-level CH_4_ emissions estimates based on aircraft measurements of air column enhancements^17^,^21^ (see Methods for description of the site-based estimate reported by Zavala-Araiza *et al*.^21^ and its use in this work.)

The site-based CH_4_ emissions estimate was nearly twice as large as a component-by-component summation based on the 2015 US Environmental Protection Agency's (EPA) national emission inventory^22^. About half of the difference was due to omission of natural gas gathering facilities in the inventory, an issue that EPA has addressed in the 2016 inventory^22^,^24^. Most of the remaining difference between the site-based emission estimate and the EPA-based component-by-component inventory was attributable to the inventory's underestimation of emissions from the region's >17,000 natural gas production sites. An important feature of the Barnett's natural gas production sites is a heavily skewed distribution of site-based CH_4_ emissions. In fact, the highest emitting 1 and 10% of sites accounted for roughly 44 and 80% of total CH_4_ production emissions, respectively^21^. Such skewed distributions have been reported in other studies of site-based and component-specific emissions across multiple regions and segments of the natural gas supply chain^2^,^3^,^4^,^5^,^11^,^18^,^25^,^26^.

We focus on the Barnett Shale region, because our analysis requires comprehensive emissions and activity data that are not currently available for other regions. The availability of rich data sets for the Barnett Shale allow us to test our hypothesis, providing a unique opportunity to compare component-based and site-based methane emission estimates^17^,^21^. Subject to data availability, our method can be applied to other regions or other industry segments. We expect that the characteristics from the emission distributions of components as well as high-emitting sites are not unique to the Barnett Shale and apply to other production regions^27^. Research underway in other regions should yield granular data sufficient to test the hypothesis presented in this work^28^,^29^,^30^.

Here we use a Monte Carlo aggregation routine to examine whether emissions from the routine operation of components present at natural gas production sites in the Barnett Shale (vented and fugitive emissions from specific equipment types or operational procedures, individually or in combination, emitting at rates sampled from emission distributions) can explain the distribution of site-based emission rates reported by Zavala-Araiza *et al*.^21^. In particular, we assess whether the predicted distribution of component emissions can potentially explain observed site-level emission rates and the presence of the highest emitting 1% of sites. We find an insufficient contribution from the components or operations that can produce high emission rates by design and conclude that this result is indicative of the existence of abnormal process conditions that create pathways for substantial unintended emissions of produced gas.

## Results

### Defining super-emitting sites

**Figure 1:**
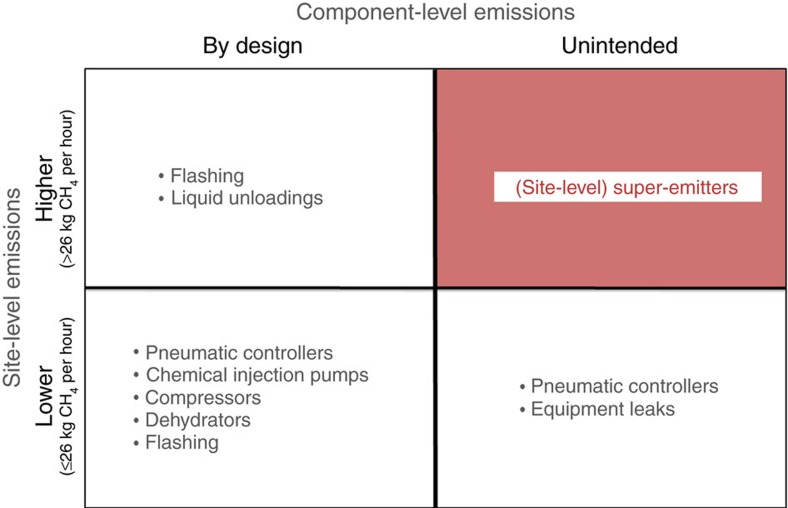
Classification of sites in terms of magnitude of emissions and component behaviour. The vertical axis classifies sites in terms of magnitude of total site-level emissions (where 26 kg CH_4_ per hour is the threshold for higher emitters). The horizontal axis classifies sites in terms of component behaviour that results in emissions (emissions by design versus unintended). The bullets in each quadrant indicate the components that were included in our component-based aggregation model; these represent all known sources of emissions on natural gas production sites in the Barnett Shale. The shaded quadrant accounts for the existence of abnormal process conditions that result in high, unintended emissions, the defining characteristic of super-emitting sites.

**Figure 2:**
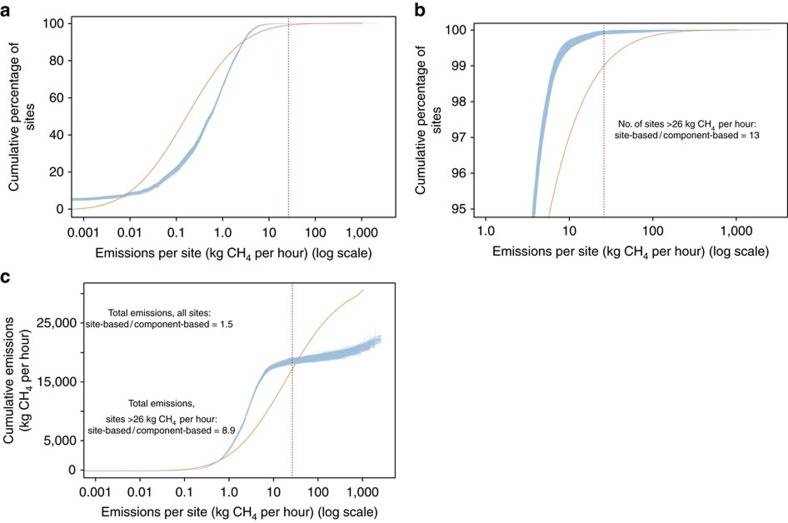
Distribution of methane emissions from production sites in the Barnett Shale. (**a**) Cumulative percentage of sites as a function of the emission rate per site. In (**b**) we show the 5% of sites with highest emissions; (**c**) cumulative emissions as a function of emission rate per site. Blue lines represent each of 10^4^ Monte Carlo iterations from the component-based aggregation reported in this work; orange lines represent the site-based results derived from Zavala-Araiza *et al*.^21^; vertical lines represent the 99th percentile of site emissions in Zavala-Araiza *et al*.^21^ (26 kg CH_4_ per hour). Inset text show ratios between site-based and component-based estimates for given metrics.

To facilitate discussion of the component-level behaviours that determine site-level emissions, we developed the classification scheme in Fig. 1. The horizontal axis in Fig. 1 distinguishes whether component emissions occur by design or are unintentional, whereas the vertical axis distinguishes total site-level emissions relative to a fixed threshold. We chose 26 kg CH_4_ per hour as the threshold for higher-emitting sites as it corresponds to the highest-emitting 1% of sites in the site-based distribution, accounting for 44% of total site emissions (Fig. 2)^21^. Although a more practical threshold for defining higher-emitting sites might include more than 1% of sites, a higher threshold simplifies the analysis and does not change the overall conclusions.

Our Monte Carlo aggregation of component emissions includes all components shown in the unshaded portions of Fig. 1. Although most modelled components emit CH_4_ by design, equipment leaks and a subset of pneumatic controllers exhibiting equipment issues produce unintended emissions. However, the resulting emission rates from these sources are not high enough to explain higher-emitting sites (that is, sites above the horizontal line in Fig. 1). Although some high-emitting operations occur by design (condensate flashing and liquid unloadings; Fig. 1, top left quadrant), they occur more than an order of magnitude less frequently than required to explain the frequency at which high site-based emissions are observed. The difference in modelled component emissions relative to the site-based estimates are largely driven by the relative absence of sites emitting >26 kg CH_4_ per hour and indicates the existence of missing sources characterized by high emissions rates. We conclude that these additional emissions are caused by abnormal process conditions that in turn lead to high, unintended emissions, the defining characteristic of super-emitting sites (Fig. 1, shaded quadrant; see below for examples).

### Quantitative evidence of the existence of super-emitters

Our component-based emission estimate is significantly lower than an independent site-based estimate. Mean total component-based emissions per site from the Monte Carlo aggregation were 1.2 kg CH_4_ per hour (95% confidence interval (CI): 1.1–1.3 kg CH_4_ per hour; Table 1), compared with the independent site-based estimate of 1.8 kg CH_4_ per hour (95% CI: 1.3–2.5 kg CH_4_ per hour)^21^. The difference between these two estimates, expressed relative to the component-based estimate, is 52% (95% CI: 9.5–110%; obtained by propagating the uncertainty of each independent estimate in quadrature) and indicates that the site-based estimate is statistically significantly higher than the component-based estimate.

To draw the conclusion that the existence of super-emitting sites results from unintended high emissions, we relied on three metrics to compare modelled component-based estimates to reported site-based distributions^21^.

First, the predicted frequency of high-emitters characteristic of the tail of the distribution is inconsistent with the observed data. On average, only 13 sites from the component-based aggregation (<0.1% of sites) exceed the 26 kg CH_4_ per hour threshold, considerably fewer than the 170 observed in the site-based distribution (Fig. 2a,b). In other words, component-based estimates do not produce enough high-emitting sites.

Our analysis shows that although pneumatic controllers, chemical injection pumps, equipment leaks, compressors and dehydrators are responsible for 83% of total component-based emissions, none of these components contribute >26 kg CH_4_ per hour to total site emissions (Table 1). By contrast, liquid unloadings and condensate flashing can produce emissions above that threshold. Liquid unloadings could result in a site having higher emissions than the maximum rate expected at any site from the observed site-based distribution (19% of simulations have maxima >1,000 kg CH_4_ per hour); however, based on self-reported frequencies of emissions from unloading events, at any given time only two sites in the region (95% CI: 0–4) have liquid unloadings. Maximum emissions from condensate flashing are ∼300 kg CH_4_ per hour, but on average only 10 events would exceed the 26 kg CH_4_ per hour threshold at any one time. In sum, the modelled frequency of liquid unloadings and high-emitting condensate flashing is insufficient to explain the number of high-emitting sites expected based on observations.

Second, all of the 10^4^ Monte Carlo component-based emission distributions underestimate cumulative emissions relative to the observed site-based distribution data (Fig. 2c). As a consequence, our component-based aggregation yields lower cumulative emissions—especially from high-emitting sites. Cumulative emissions from the site-based distribution (31,000 kg CH_4_ per hour 95% CI: 22,000–43,000 kg CH_4_ per hour) are 1.5 times larger than those from the component-based aggregation (20,000 kg CH_4_ per hour 95% CI: 19,000–22,000 kg CH_4_ per hour). The disparity is especially pronounced for high-emitting sites. The highest emitting 1% of sites in the observed site-based distribution have cumulative emissions nine times larger than the component-based aggregation would predict (14,000 kg CH_4_ per hour versus 1,500 kg CH_4_ per hour, 95% CI: 460–3,800 kg CH_4_ per hour). The relationships between component and site-based emissions characteristics in Fig. 2 are further examined in the Supplementary Information using a metric of disproportionality^31^, affirming the insufficient contribution from high-emitting sites in the component-based aggregation (Supplementary Note 1 and Supplementary Fig. 1).

The component-based aggregation produces reasonable agreement with the observed site-based emission distribution for the lowest emitting ∼90% of sites (Fig. 2a). The small overestimation of cumulative emissions in the component-based aggregation for these sites could be an artefact of activity factors derived from the EPA Greenhouse Gas Reporting Program (GHGRP) being unrepresentative for less complex sites with lower gas production rates (that is, less equipment per site than modelled). As 80% of cumulative site-based emissions comes from the last 10% of the distribution (Fig. 2c), the effect of overestimating emissions from low-emitting sites has little impact on our overall conclusions.

Third, 30% of Barnett production sites have instantaneous emission rates that exceed 1% of the natural gas they produce and account for 70% of total emissions (21,000 kg CH_4_ per hour)^21^. Previous work showed that many sites with high instantaneous loss rates (emissions equal to 2–50% of gas production) also had high absolute emissions (20–300 kg CH_4_ per hour); such sites were classified as ‘functional super-emitters'^25^.

Our component-based aggregation predicts that 47% of sites would have loss rates >1%, somewhat higher than the 30% predicted by the site-based distribution. However, cumulative emissions from sites with component-based loss rates >1% are only 13,000 kg CH_4_ per hour (95% CI: 12,000–16,000 kg CH_4_ per hour), significantly lower than for the observed site-based distribution. (Supplementary Fig. 1). The larger fraction of sites with component-based loss rates >1% is due mainly to our model's over prediction of emissions for the lowest-emitting 90% of sites. In summary, the component-based aggregation under predicts the number of sites exhibiting both high absolute emissions and high proportional loss rates.

## Discussion

The inability of routine operating conditions to explain the sources of CH_4_ at high-emitting sites reveals the existence of super-emitters: sites with abnormal process conditions. The work reported here shows that the routine emissions of components observed at natural gas production sites fail to explain critical characteristics of the independent emission distribution determined from site-based measurements, in particular the disproportionate contribution of emissions from high-emitting sites, which also have high loss rates. Based on the differences between our predicted component-based aggregation and observed site-based results, we infer that the missing emissions are most likely to be due to abnormal process conditions causing sites to become super-emitters. These super-emitting sites account for approximately one-third of total emissions from natural gas production sites.

Based on direct observations from recent field campaigns on production and midstream sites^2^,^5^,^18^,^24^,^25^,^32^,^33^, examples of abnormal process conditions—which could be persistent or episodic—could include: failures of tank control systems, malfunctions upstream of the point of emissions (for example, stuck separator dump valve resulting in produced gas venting from tanks), design failures (for example, vortexing or gas entrainment during separator liquid dumps) and equipment or process issues (for example, over-pressured separators, malfunctioning or improperly operated dehydrators or compressors). As the causes of high unintended emissions may not reside within the emitting component itself, rather upstream or even downstream in some cases, and are inherently difficult to measure, it is not surprising that component-based inventories do not account for these emissions and thus underestimate overall emissions at the site level. Future work should focus on measurement and characterization of the variety of high, unintended emissions at production sites.

Additional evidence for the causes of high, unintended emissions hypothesized above is evident in the results of an aerial infrared camera survey of over 8,000 natural gas-producing sites in seven US regions, including the Barnett, and representing ∼1% of all well pads^32^. That study found that emissions from tank vents and hatches accounted for roughly 90% of all detected hydrocarbon sources emitting >3–10 kg per hour (Supplementary Note 2). Other sources observed included separator pressure relief valves, dehydrators and flares. The observed frequency of tanks as high-emitting sources in the Barnett far exceeded what would be expected from well-controlled condensate flashing^28^. The independent aerial survey results, coupled with the low expected frequency of liquid unloadings events in the Barnett, point to the existence of conditions leading to high emissions that probably manifest as venting from tanks but that are not explained by the routine behaviour of components, including condensate/oil flashing or liquid unloadings (Table 1 and Supplementary Fig. 2). Although the survey results described above did not yield an emission estimate, the present work suggests that unintended tank emissions are significant and consistent with recent observations at gathering facilities^5^.

The abnormal process conditions that define super-emitting sites are characterized by stochastic, spatio-temporally dynamic behaviour^28^. Thus, specific sites could be affected by abnormal conditions resulting in their being a super-emitting site at varying points in time. As a consequence, rather than looking to control emissions from a few sites, minimizing emissions requires monitoring approaches that enable efficient and timely responses to the unpredictable nature of when and where a super-emitter will be located.

Frequent or better yet continuous site-level monitoring of emissions or process conditions would reduce the duration of super-emitting behaviour. Moreover, such monitoring should produce accumulated learning, which in turn provides the knowledge to improve equipment, system design and operations that would reduce the frequency of large emission events.

## Methods

### Monte Carlo aggregation of component-based emissions

We developed a Monte Carlo aggregation routine to model the distribution of CH_4_ emissions expected from the routine operation of the population of natural gas-producing sites in the Barnett Shale region (*N*=17,400). CH_4_ emissions from natural gas production sites come from a discrete set of components (Table 1) as follows: pneumatic controllers (used to control the operation of mechanical devices or processes on site); chemical injection pumps; leaks from valves, flanges, fittings and other equipment; compression systems (compressors and compressor engines with fugitive, vented and exhaust emissions); dehydrators; and flashing from condensate/oil or produced water. In addition, at some sites there are occasional operational practices that vent natural gas, for example, liquid unloadings (clearing the wellbore of accumulated liquid), compressor blowdowns and compressor start-ups. Hereafter, we use the term components to refer to all routine sources of emissions at gas production sites including both equipment and operational procedures. We use site-specific data on the number of wells and production of natural gas, condensate/oil and water, to estimate emissions from each of eight components and then aggregate those to estimate total site-level emissions. See Supplementary Fig. 3 for a flow chart of the Monte Carlo aggregation process.

For discrete components such as pneumatic controllers, chemical injection pumps and equipment leaks, we sample from reported measurement distributions to assign emissions to each component at each site. We acknowledge that part of the uncertainty in our methods includes the possibility of higher emissions from these component types that are not captured by the sampled data sets, although for the following reasons we believe any effect would probably be small. The component measurement distributions from which we sample are skewed (for example, 13% of the measured pneumatic controllers account for 88% of the emissions, 10% of the chemical injection pumps account for 50% of the emissions and 11% of equipment leaks accounted for 70% of emissions); hence, our model partially captures the effect of components with fat tails and unintended emissions. The possible effect of high emitters outside the measured distributions from which we sample is constrained by the fact that Lyon *et al*.^32^ did not observe these component types to be high emitters (>3–10 kg per hour). In Supplementary Note 3 we fit the distribution of chemical injection pump measurements to several functions characteristic of skewed distributions. We use the best-fitting function to assign emissions in our model and conclude that even though maximum emissions from pumps can increase slightly, they do not modify our conclusions.

We chose not to sample from functional fits to the measurement data for simplicity and because the results were not significantly affected. In addition, fitting to probability distribution functions (pdfs) carry its own set of assumptions, uncertainties and challenges related to sample sizes and characteristics of the population of discrete components (for example, equipment leaks linked to different processes and operating pressures or pneumatic controllers servicing heterogeneous applications).

Emissions from liquid storage tanks can result from routine operations or from unintended conditions such as stuck separator dump valves. Our analysis considers two types of routine emissions released from tanks: flashing from condensate/oil or produced water and liquid unloadings. We exclude unintended conditions from the component-based aggregation, as they are not characteristic of routine operations and are largely excluded from current emission inventories.

We estimated emissions from condensate/oil flashing using site-specific production rates and published emission measurements from the Barnett Shale. We assumed an intermittent flash emission profile for most sites; a continuous profile was assumed for sites where the liquid production rate exceeded the assumed intermittent separator dump rate (see below for description of the process of liquid dumps from separator and resulting tank flashing). Emissions from the highest condensate/oil producing sites (that is, those accounting for 60% of total condensate/oil production) were assumed to be controlled. We tested the effect of applying controls to alternative fractions of production (20, 75 and 90%). Flashing from produced water was modelled in the same way, except that we used a water-specific emission rate and assumed no emissions controls.

For liquid unloadings, we used EPA GHGRP^23^ data to determine an expected frequency of two unloading events that vent during any given hour in the Barnett (95% CI of 0–4 events per hour) and sampled from reported emission distributions (distinguishing between wells with and without plungers). We modelled emissions from compressor start-ups and blowdowns in a similar way; these were included along with the emissions from routine compression system operations. Well completion events are excluded from our analysis, because they did not occur at sites used to derive the site-based distributions^20^,^21^,^25^.

Aggregating component emissions for each site creates an emission distribution for the population of sites. We repeated this process 10^4^ times to account for the variability in the existence of specific components at each site and the expected stochastic nature of their corresponding emissions. Finally, we compare our model results with the independent site-based emission distribution reported by Zavala-Araiza *et al*.^21^

To compare our results with Zavala-Araiza *et al*.^21^ our analysis seeks to characterize emissions from the population of natural gas producing sites in the Barnett Shale production region (North Central Texas) with reported gas production, in 2013. Data for each well in the Barnett were obtained from DI Desktop^34^ and clustered into sites as described in Zavala-Araiza *et al*.^25^ For each site, we know the number of wells, age, oil (condensate) production, water production, and natural gas production (see Supplementary Note 4 for discussion of representativeness of site-based measurements from Zavala-Araiza *et al*.^21^).

The total count of 17,400 sites excludes 84 sites classified as oil sites: 77 sites containing ≥6 oil wells with initial production before 1990 (not likely to be multi-well pads characteristic of shale development) and 7 newer sites containing 11–68 wells but which appeared on GoogleEarth to be distributed oil wells and not multi-well production sites. These sites are an artifact resulting from DI Desktop's assignment of identical coordinates to multiple oil wells; even if these sites actually existed, their characteristics would be unrepresentative of the natural gas production sites that comprise the reported distributions of site-based measurements and our component-based simulations. As we used the number of wells and production rates as drivers to estimate component counts and emissions at each site, including these unrealistic oil sites would bias results. The excluded sites account for a total of 1,360 wells (5% of total gas producing wells in the region), total gas production of 2,860 Mcf per day (<0.06% of total gas production), total oil production of 5,660 bbl per day (14.5% of oil/condensate production) and total water production of 505, 500 bbl per day (39% of total water production). After this correction is applied, the total number of wells at the 17,400 sites is 25,700, with a mean of 1.5 wells per site (median=1 well per site; range of 1–22 wells per site). Supplementary Fig. 4 shows the distribution of wells per site for the 17,400 natural gas production sites.

### Site-based emission estimates

In this work, we compare our component-based aggregation of emissions with the pdf derived by Zavala-Araiza *et al*.^21^ for production sites. Zavala-Araiza *et al*.^21^ reported a site-based emission distribution using a statistical estimator approach, to develop an integrated distribution from a systematic sample and a high-emitter biased sample of ground-based, downwind measurements of facility-wide emissions from gas production sites (discussion of the representativeness of the data used in Zavala-Araiza *et al*.^21^ to develop the site-based distribution and the plausibility of site emissions between 300–1,000 kg CH_4_ per hour is provided in Supplementary Note 2)^21^.

Specifically, Zavala-Araiza *et al*.^21^ developed an emissions pdf that describes the expected emissions from a population of production sites by integrating a data set of CH_4_ measurements of 186 Barnett Shale production sites, where the sampling team quasi-randomly selected the sites to be measured (systematic sample)^35^, and a data set of CH_4_ measurements of 81 Barnett Shale production sites, where two research teams were explicitly looking for high emitters (high-emitter biased sample)^9^,^10^. They modelled the high-emitter bias (for example, power law, Gaussian plume theory), providing a method that seamlessly derives a continuous pdf that is representative of the entire population of sites. The derived pdf follows a lognormal distribution with parameters *μ*=−1.8 (CI: −2.1,−1.5) and *σ*=2.2 (CI: 2.0, 2.4), which can be used to obtain an average site-level emission factor (EF) of 1.8 kg CH_4_ per hour (CI: 1.3, 2.5):


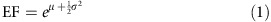


### Methods for pneumatic controllers

Supplementary Fig. 5 shows a flow chart summarizing the methodology followed to estimate emissions from pneumatic controllers. Allen *et al*.^2^ report 377 measurements of emissions from pneumatic controllers. Their results show significant differences in emission rates for the different controller applications. To account for this variability, we classified the data set of pneumatic controllers emissions into the main controller applications: separator, process heater, compressor, dehydrator, wellhead and plunger lift. This classification also allows the count of pneumatic controllers in our simulation to directly track with the equipment on the sites. With this classification scheme we are able to simulate the diversity of emission rates across the range of pneumatic controller applications. In addition, this approach preserves the variability in the distribution of pneumatic controllers per site in the Barnett Shale. We acknowledge that this approach relies on a national data set. As discussed in Allen *et al*.^2^, there are important regional differences in terms of emission rates from pneumatic controllers; this could be driven by regulations on pneumatic controllers in some regions. Using a regional subset applicable to the Barnett would probably yield higher emissions from pneumatics; however, we would lose the ability to separate controllers into their application (because of the small regional sample sizes in the original data set). As this assumption does not change our overall conclusion, we decided to preserve the variability in the distribution of pneumatic controllers per site and use the entire national data set. We estimated the average number of pneumatic controllers per application from the equipment counts per site reported by Allen *et al*.^2^ (for example, number of pneumatic controllers per compressor). In turn, we used the GHGRP^23^ to derive regional activity factors for the counts of different equipment types per well (for example, number of compressors per well). By combining the GHGRP equipment counts per well with the counts of pneumatic controllers per equipment type and multiplying by the total number of wells in the region (25,700 wells in the Barnett Shale production region), we estimated the total count of pneumatic controllers (by equipment/application). We then assigned these pneumatic controllers to specific sites and sampled from the reported measurements in Allen *et al*.^2^ to assign emission rates. See below for description of how compressors and dehydrators are specifically assigned to sites.

For those cases where the activity factor of equipment per well or pneumatic controllers per application is a fraction above one (for example, 4.3 pneumatic controllers per compressor), we first assign the integer part of the activity factor to all sites (for example, 4 pneumatic controllers to each compressor), then we estimate the total count that results from the fractional part of the activity data (for example, 0.3 times the total number of compressors) and consequently assign those additional controllers to randomly chosen sites with the relevant equipment type (for example, compressors).

For pneumatic controllers assigned to plunger-lift systems, data from Allen *et al*.^2^ shows a probability of 0.94 of having a pneumatic controller for wells with plunger lift. We assumed one pneumatic controller per well with plunger lift, and that 40% of the wells in the region have plunger lifts^23^.

For the case of pneumatic controllers assigned to separators, we did not use the GHGRP activity data because of the inherent differences between sites with and without oil production. As sites without oil production are expected to have a 2-phase separator (water–gas), we assign one separator per well and one pneumatic controller per separator (12,600 sites; 72% of sites).

For sites with oil production, we assigned one three-phase separator per well (oil–water–gas), with two pneumatic controllers per separator. The GHGRP shows a ratio of 1.06 separators per well (for all wells). We attribute the fraction of 0.06 to the use of a second lower-pressure separator on some sites. Consequently, we distributed a corresponding number of additional two-phase separators (oil–gas) with a single pneumatic controller to randomly selected sites with reported oil production.

In summary, 72% of sites with no condensate/oil production get one 2-phase separator per well and 1 pneumatic controller per separator (totalling 18,250 pneumatic controllers). The remaining 28% of sites get one 3-phase separator per well and 2 pneumatic controllers per separator (totaling 14,900 pneumatic controllers), and finally 450 additional 2-pahse separators with 1 pneumatic controller are each randomly assigned to the latter subset of sites.

Supplementary Table 1 summarizes the activity factors and emissions factors for the different applications to which we assign pneumatic controllers. Supplementary Fig. 6 shows the distribution of emissions from pneumatic controllers.

### Methods for chemical injection pumps

Supplementary Fig. 7 shows a flow chart summarizing the methodology followed to estimate emissions from chemical injection pumps. Allen *et al*.^1^ report measurements of CH_4_ emissions for 62 chemical injection pumps. The average EF is 0.22 kg CH_4_ per hour per pump (range: 0.002–2.3 kg CH_4_ per hour). We derived a GHGRP^23^ activity factor (for the Barnett Shale) of 0.54 chemical injection pumps per well, to estimate the total number of chemical injection pumps in the region. For a total of 25,700 wells in the region, we estimated 13,900 gas-driven chemical injection pumps. Consequently, we randomly selected the sites that are assigned chemical injection pumps (if a site gets selected, all the wells on that site are assigned pumps) and assigned an EF to each pump by sampling from the distribution of measured emission rates^1^. Supplementary Fig. 8 shows the distribution of emissions from chemical injection pumps.

In Supplementary Note 3 we fit the measurements from chemical injection pumps to different pdfs. We conclude that even when the fitted distribution would lead to sampling emissions higher than the ones measured, this does not substantially affect our results.

### Methods for equipment leaks

Supplementary Fig. 9 shows a flow chart summarizing the methodology followed to estimate emissions from equipment leaks. Allen *et al*.^1^ measured a total of 278 equipment leaks detected by infrared camera surveys at 150 natural gas producing sites in the United States (total number of wells=478). The average EF is 0.12 kg CH_4_ per hour per leak (range: 0.0–5.6 kg CH_4_ per hour). They report an EF of equipment leaks per well calculated as follows:


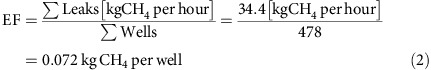


As observed in equation (2), embedded in the EF is an implicit assumption of the average number of wells per site (3.2 wells per site) from the specific sites where measurements took place. For the Barnett Shale, there are 17,390 sites with 25,700 wells, resulting in an average of 1.5 wells per site. Because of the lower average well count per site in the Barnett compared wth the population of sites sampled in Allen *et al*.^1^, if we used Allen *et al*.'s per well EF, we would potentially underestimate emissions from equipment leaks.

To correct for this, we classified the sites where Allen *et al*.^1^ took measurements into cohorts based on the number of wells at each site. For each cohort we can observe a distribution of the number of measured leaks (Supplementary Table 2). Although Allen *et al*.'s data set is insufficient to draw definitive conclusions about the relationship of leaks per site and well count, it is noteworthy that leaks per site tend to increase with the number of wells, but appear to level off (and leaks per well would tend to decrease). Our model used the number of wells on each site to randomly select a number of leaks from Allen *et al*.'s distribution of sites in the corresponding cohort (Supplementary Table 2). Finally, we sampled from the distribution of emissions per leak to populate the leaks assigned to each site. Supplementary Fig. 10 shows the distribution of emissions at natural gas production sites from equipment leaks.

### Methods for compression systems

Supplementary Fig. 11 shows a flow chart summarizing the methodology followed to estimate emissions from compressors. There are four major sources of emissions related to compression systems on production sites: fugitive emissions, engine exhaust emissions, compressor start-ups and compressor blowdowns.

To determine the total number of compressors at natural gas production sites in the region, we derived from the GHGRP^23^ an activity factor (for the Barnett Shale) of 0.14 compressors per well. For a total number of 25,700 wells in the region we estimated 3,600 compressors. We determined a distribution for the number of compressors per production site using the Texas Commission on Environmental Quality (TCEQ)'s 2009 Barnett Shale Special Emissions Inventory (BSEI)^36^. For the subset of sites in the inventory that reported at least one compressor, the average was 1.2 compressors per site with a range of 1 to 5. We used this distribution to assign a specific number of compressors to randomly selected sites until we reached the total count of compressors in the region. The number of compressors at each site never exceeded the well count. For each site, we then estimated total compressor emissions (considering fugitives, engine exhaust, start-ups and blowdowns).

There is limited data on fugitive emissions from compression systems at production sites. We used a central EF of 0.24 kg CH_4_ per hour (95% CI: 0.16–0.32 kg CH_4_ per hour)^22^ based on the EPA GHGI EF; for each compressor (based on the EPA GHGI EF), we sampled from a normal distribution with those characteristics.

For the case of engine exhaust, the TCEQ BSEI^36^ reports engine horsepower data (*N*=1,100; mean=156 HP; range of 10–1,340 HP). We sampled with replacement from this distribution to assign a specific horsepower to each compressor. We estimated a central emission rate for CH_4_ in engine exhaust based on manufacturer specifications for the Caterpillar 3306, which represents ∼75% of reported engines in the BSEI, augmented by 25% to reflect real-world performance. We also used a distribution of ±50% around this central value to account for potential variability in operation and maintenance. Consequently, we use a central EF of 0.0013, kg CH_4_ per hour per HP-hour (95% CI: 0.0005–0.002 kg CH_4_ per hour per HP-hour), sampling from a normal distribution with those characteristics. For comparison, the AP-42 EF for comparably sized rich-burn engines is 0.00084, kg per HP-hour (95% of well site compressors in the BSEI are rich-burn)^37^.

Compressor start-ups and blowdowns are modelled as episodic emissions. We determine the expected frequency of events during any given hour and then sampled from their respective distributions of EFs.

We assumed that a compressor start-up could take place any given working hour of the year (4,380 working hours per year). For an average frequency of 13 start-ups per compressor per year^37^ and 3,600 compressors in the region, the total number of events per year would be 46,800. Assuming a duration of 1 h, the average frequency of compressor start-ups during any given working hour of the year equals 11.

We sampled (with replacement) from the 4,380 working hours of the year, until we assigned the total events per year. Consequently, we determined the 95% CI of the expected frequency of events per hour: 10 (95% CI: 4–17).

In each Monte Carlo run, first we sample a frequency of compressor start-up events from the normal distribution generated as a result of the bootstrapping process. Second, from the subset of sites where compressors were previously assigned, we randomly select the sites that get the compressor start-up events. For the selected compressor, we eliminate the assigned exhaust and fugitive emissions. Finally, we assign an emission rate by sampling from a normal distribution with an average EF of 12 kg CH_4_ per hour per start-up (95% CI: 6.1–18 kg CH_4_ per hour)^38^.

For compressor blowdowns, there is an average frequency of 11 blowdowns per compressor per year^38^ and for 3,600 compressors in the region the total number of event per year would be 39,600. If we assume duration of 1 h, the average frequency of compressor blowdowns for any given working hour of the year would be 9.0.

Using a bootstrap method, we sample (with replacement) from the 4,380 working hours of the year, until we assign the total number of events per year. From this process we determine the 95% CI of the expected frequency of events per hour: 9.0 (95% CI: 4–15).

In each Monte Carlo run we followed a similar procedure to the start-up events, with the difference that we assign an emission rate to each blowdown event by sampling from a normal distribution with an average emissions factor of 6.5 kg CH_4_ per hour per blowdown (95% CI: 3.3–9.8 kg CH_4_ per hour)^38^. Supplementary Fig. 12 shows the resulting distribution of emissions from compressors.

### Methods for oil/condensate flashing

Flashing is a rapid process that occurs when a liquid that contains dissolved gases is transferred from a pressurized separator to a storage tank at atmospheric pressure^39^. Although several models exist to estimate daily or annual flashing emissions, there is little published data that can inform the simulation of time-dependent flashing emissions. We developed a probabilistic model to estimate the probable range of flashing emissions from Barnett natural gas-producing sites at any given time. We need to characterize a population's expected behaviour at a typical point in time, to make comparisons with site-based observations from mobile surveys^21^. Here we describe the assumptions we used to simulate flashing emissions in the Barnett Shale. Our assumptions are based on the best available Barnett-specific data and are chosen conservatively so that they may err on the side of higher emission rates. We apply an additional enhancement factor to account for the possibility of site-specific conditions that could lead to even higher emissions at some sites. As a result, we conclude that our model predictions are a reasonable upper bound for the contribution of flash emissions from condensate/oil production systems, operating as designed, in the Barnett Shale.

We used site-specific production rates of condensate/oil to determine whether venting of flash gas at individual sites is continuous or intermittent. Supplementary Fig. 13 shows the distribution of condensate/oil production rates at natural gas-producing sites in the Barnett Shale. Only 28% of natural gas producing sites had any reported condensate/oil production (range of 0.1–967 bbl per day, median of 1.2 bbl per day and average of 8.0 bbl per day). If the condensate/oil production rate at a site exceeds the maximum rate of dumping from separators into liquid storage tanks (see below, typically >140 bbl per day), we assume that emissions from oil flashing occur continuously.

In each Monte Carlo run, we estimate this maximum rate of dumping from the separators, equivalent to a continuous dump threshold by sampling from a distribution of dump volumes and dump durations. As a result, continuous flashing is predicted to occur at 13 sites (95% CI: 3–32 sites). If the condensate/oil production rate is smaller than the continuous dump threshold, we estimate the ratio of the condensate/oil production rate to the continuous dump threshold. The average ratio represents the average fraction of sites that would have intermittent flashing events at any given time. Intermittent venting is thus predicted to occur at 132 sites at any given instant (95% CI: 70–203 sites).

There is limited publicly available data about the characteristics of liquid dumps from separators into tanks (dump volumes, frequency and duration). Based on input from experts on the design and operation of natural gas production sites, we assumed that about 0.05–0.1 bbl of liquid are dumped per separator dump cycle (∼2–4 gallons per dump). Visual inspection of the time series of discretely actuating intermittent separator level controllers in a study on pneumatic controller emissions indicate some intermittent controllers actuated as much as ∼80% of time, suggesting nearly continuous dumping.^2^ Another study reported that a tank in the Denver–Julesburg basin producing 29 bbl per day of condensate flashed ten times in 20 min, with primary flashing events lasting from ∼30 to ∼120 s (ref. 8). Based on this limited data we modelled the duration of condensate/oil dumps using a distribution centred around 30 s (95% CI of 15–45 s). The short interval was intended to lead to an upper bound of emission rates from high-emitting flash events.

We estimated the magnitude of flash emission rates from condensate/oil produced in the Barnett Shale region using the results of direct vent gas measurements reported in a survey of tanks in the region (the so-called ‘HARC study' after the sponsoring organization, the Houston Advanced Research Center)^40^. Results for 3 tanks (17, 25 and 26) were excluded from the full data set due to abnormally high emission rates—representing more than 50% of the pre-flashed liquid—potentially caused by equipment failures such as a stuck valve or measurement error^33^. (At the end of this section, we discuss the potential contribution of such abnormally high-emitting tanks to site-level emissions.) Tank venting emissions reported in the HARC study include contributions from flashing, as well as working and breathing losses occurring over the 24 h diurnal cycle of an individual measurement. Given the high volatility of dissolved CH_4_ at ambient conditions, the effect of working and breathing losses on estimates of CH_4_ flashing are expected to be small. We did not use predictive models to estimate flash emissions due to a lack of site-specific data and such models' generally poor correlation with measurements^41^. Limitations of flash emissions models include limited input ranges (typical operating conditions such as separator pressure often exceed the model's range) and their requirement for input parameters such as pressurized liquid composition that are difficult to sample and analyse in reproducible ways.

In each Monte Carlo run, each site is randomly assigned one of the 20 filtered CH_4_ emission rates from the HARC study (range of 17–412 scf per barrel, with a mean of 76 scf per barrel). To place this distribution into context, we used the Vazquez–Beggs equation to estimate flashing emissions^42^. Using the highest combination of API gravity (61) and separator pressure (200 psig) from the HARC data set (Supplementary Table 3), estimated flashing emissions using the Vazquez–Beggs equation would be 150 scf per barrel of total flash gas or 51 scf CH_4_ per barrel (Assuming that the fraction of methane in the flash gas is similar to the one in the HARC data set, ∼33%).

As our methodology assigns flash EFs randomly to sites without consideration of site-specific process parameters such as separator pressure, our estimates represent an upper bound for the 450 wells (9% of wells with condensate/oil production) that may use a second, lower pressure separator.

Although limited in size, the HARC study is the largest data set of tank flash measurements for the Barnett Shale region. The measurements in the HARC study cover both condensate and oil tanks, as well as a large range of API gravities and separator pressures. The populations sampled and results are largely consistent with two smaller studies in the region, although the HARC study sampled lower-producing condensate sites (Supplementary Table 3). The size of data sets characterizing flash emissions is limited because of the high cost of individual measurements (typically representing a 24 h time series and possibly involving multiple emission locations on the tank such as hatches and pressure relief valves) and difficulties in obtaining site access. The results of the HARC study also compare reasonably well with limited tank measurements reported in the GHGRP (Supplementary Table 4)^23^ and the EPA Oil/Gas Estimation Tool (205 scf per barrel of total vent gas and 93 scf CH_4_ per barrel for the Bend Arch–Fort Worth Basin)^43^.

The use of capture or control equipment could also affect condensate flashing emissions. Tank emission control devices can include flares, combustors (enclosed flares) and vapour recovery units. In October 2013 (when the site-level measurements to which we compare our results were made), tank emissions from a subset of sites in the Barnett Shale were either captured or controlled due to federal and state requirements. It is also possible that operators had installed vapour recovery units at high oil-producing well sites for purely economic reasons. The federal New Source Performance Standard Subpart OOOO requires certain tanks with the potential to emit ≥6 tons per year volatile organic compounds (VOC) to install control devices. Subpart OOOO applies to individual tanks installed or modified after 2011, with varying compliance dates ranging from 2013 to 2015, depending on the exact date of installation or modification^44^. A Texas state regulation limits total site-wide VOC emissions from oil and gas production sites (that is, not just tank emissions) to 25 tons per year^45^. For reference, 6 tons per year of VOC corresponds to ∼3 bbl per day of condensate production in the Barnett Shale, using an EF of 9.8 lb VOC per barrel (25 tons per year VOC would correspond to ∼14 bbl per day of Barnett condensate)^46^.

The shifting regulatory landscape makes it challenging to estimate the level of control at the specific point in time when site-level measurements were made (October 2013). A review of results from a mandatory survey of Barnett Shale operators concluded that 12% of surveyed oil production in the Barnett region in 2009 was controlled^33^. A separate, voluntary survey of operators in other parts of Texas found that 91% of surveyed production in 2011 was controlled, suggesting the 2009 Barnett value of 12% may not reflect practices in more recent years (no operators in the Barnett were surveyed in the voluntary survey)^33^. An analysis of sites potentially subject to Subpart OOOO estimated that an additional 4–6% of Barnett oil/condensate production would have been controlled in 2013 (that is, additional to the percentage already controlled before Subpart OOOO implementation)^46^. Based on these three surveys, we conclude that 20% represents a lower limit for the amount of Barnett Shale oil/condensate production that was controlled in October 2013.

A more realistic central estimate of the controlled fraction of oil/condensate production can be obtained by examining site-specific production rates. Sites with condensate production >20 bbl per day would probably have potential emissions in excess of the Texas regulatory limit of 25 tpy VOC for oil and gas production facilities (20 bbl per day yields about 36 tpy VOC)^46^. In October 2013, ∼350 natural gas-producing sites in the Barnett Shale also produced >20 bbl per day of condensate; total production of these sites was 66% of the total oil production at all 17,390 gas-producing sites in the region (∼22,200 versus ∼33,400 bbl per day).

Based on our estimate that at least 66% of Barnett condensate/oil production occurs at sites with potential VOC emissions above the Texas regulatory limit of 25 tpy VOC, we assume in our Monte Carlo analysis that 60% of production is controlled. The lower control factor would account for the possibility that not all sites comply with control requirements. We also ran the model using our estimated 20% lower limit of the fraction of production that is controlled (75 and 90% controls were also modelled). Results under different control scenarios are summarized in Supplementary Table 5.

To simulate the effect of tank emission controls in our model, we ranked sites by oil/condensate production and assigned controls to sites in order of decreasing production until we reach 60% of total production. Approximately 5% sites with oil/condensate production were thus assigned controls (Supplementary Table 6). Sites with controlled emissions are assumed to emit 2% of their potential flash emissions (we assume those sites would have a flare or combustor with 98% combustion efficiency). Uncontrolled sites are assumed to vent their total potential emissions.

For intermittent flashing, our modelling approach assumes that flashing emissions occur as the result of individual separator dumps of 0.05–0.1 bbl and venting duration of 15–45 s. Our EFs (Supplementary Table 3) are based on emission measurements at sites with a range of separator pressures and liquid composition (API gravity). In reality, it is possible that flash events could occur outside of these conditions, leading to higher (or lower) emission rates than we model. It is also possible for multiple separators to be dumping into the same tank battery with a shared vapour space, resulting in a higher effective dump volume. There is also uncertainty about the time profile for the venting of flash gas resulting from individual or combined separator dumps. By definition, the vent duration must be equal to or longer than the duration of the corresponding liquid dump. For tanks not connected to vent capture or control systems, the vent duration will depend on whether tank hatches are open or closed and (for tanks with closed hatches) the pressure settings and maximum flow rates of a tank battery's pressure relief valve(s). To account for these uncertainties (vent duration and dump volume), in each Monte Carlo iteration of our model, we randomly assign an enhancement factor of 1–2 × at ∼50% of sites to account for the possibility of higher emission rates from flashing (average enhancement factor: 1.4, 95% CI: 1.3–1.5).

Besides infrequent liquid unloadings events in the Barnett, condensate/oil flashing is the only modelled component that can directly cause emissions at a level found among the fat tail of emissions. Regrettably, there is limited published data about the temporal patterns of flashing emissions. We simulated flashing with continuous and intermittent temporal profiles, assuming that 60% of total condensate/oil production is controlled (we also ran sensitivity tests assuming different levels of control device deployment, all at an assumed control effectiveness of 98%). Our model predicts that at any given time 143 sites (95% CI: 74–240 sites) would have flash emissions, with 13 sites (95% CI: 3–32) venting continuously and 130 sites (95% CI: 70–200) venting intermittently. Such a contribution from flashing is insufficient to explain the observed frequency of super-emitters (only 10 sites would have condensate tank flashing emissions >26 kg CH_4_ per hour; Table 1). Assuming a 20% control technology deployment (representing the lower bound of expected controls in the region), total emissions would only increase by ∼4%, with the number of sites in the fat tail (19 sites) still well below what is expected from the site-based analysis.

As discussed above, we excluded from our sampled distribution of flash emission rates 3 of 20 measurements of Barnett condensate and oil tanks reported in the HARC data set^40^, because they appeared to indicate abnormal conditions or measurement error^33^. The three excluded tanks had the highest reported VOC emission rates among all tanks sampled (2–19 times higher than the next highest value); 2 of the 3 excluded tanks also had the highest CH_4_ emission rates (∼3 times the next highest value). The third excluded tank (25) exhibited an unrealistically low CH_4_ mole fraction (<0.1% by weight of sampled vent gas), suggesting a sampling or analytical artefact. We conducted a thought experiment to assess the implications that the emissions from 15% of the tanks sampled in the Barnett may have exhibited abnormal process conditions.

We estimated maximum potential emission rates due to abnormal process conditions by using the CH_4_ emission rates reported for tanks 17 and 26 (1,220 and 1,271 scf CH_4_ produced in 24 h from reported condensate production of 1 and 2 barrels, respectively.) If the emissions occurred continuously, the rate would be ∼1 kg CH_4_ per hour, which is not high enough to explain super-emitting sites (emissions >26 kg CH_4_ per hour). On the other hand, if emissions were limited to discrete liquid dump cycles consistent with our modelling assumptions (0.1 barrels per dump, vent duration of 30 s), then emission rates in the range of 150–300 kg CH_4_ per hour could be expected. Such intermittent rates are high enough to explain emissions from sites in the high end of the site-based distribution. Consequently, observations from the HARC data set provide suggestive evidence for the existence and potential magnitude of super-emitting sites (Fig. 1). Possible causes could include, for example, gas entrainment due to vortexing through the separator outlet or an improperly set liquid level set point.)

We also note the existence of tank vent controls on some sites may lead to a smaller number of sites with actual emissions above the 26 kg CH_4_ per hour threshold. In any case, we conclude from this thought experiment that abnormal separator/tank behaviours evident in the HARC study could lead some sites to be super-emitters; however, these behaviours do not explain the entire shortfall of sites >26 kg CH_4_ per hour at the fraction of 15% abnormal oil/condensate tanks observed in the HARC study (similar behaviour could apply to water tanks; see below). The percentage of tanks with abnormal process issues may be higher in the Barnett than observed in the HARC study and/or some super-emitters may have other causes. A recent survey found that tanks represented 90% of high-emitting source on oil and gas production sites, which strongly suggests that tanks are the main emission point of the vast majority of sites with >26 kg CH_4_ per hour^32^. Supplementary Fig. 14 shows a flow chart summarizing the methodology followed to estimate emissions from condensate tank flashing.

### Methods for water flashing

With two exceptions, we model emissions from water flashing in the same way we modelled flashing from condensate/oil production. First, we use an EF of 2.6 and 0.74 scf CH_4_ per bbl for gas wells and oil wells, respectively^43^. Second, we assume emission from water tanks are uncontrolled. Based on limited evidence from air permits, it is likely to be that some water tanks at some sites are manifolded together with controlled condensate tanks. Therefore, this assumption will lead to somewhat higher emissions from water flashing than would be expected in practice. We simulated flashing with continuous and intermittent temporal profiles; our model predicts that at any given time 2,000 sites (95% CI: 1,200–2,800 sites) would have flash emissions, with 700 sites (95% CI: 300–1,200) venting continuously and 1,300 sites (95% CI: 900–1,600) venting intermittently.

We also note that if abnormal separator/tank behaviour can cause intermittent, high emission rates related to water dumps similar to those observed from condensate tanks by the HARC study, then a fraction of sites with this issue could account for the number of sites >26 kg CH_4_ per hour. If 10% of the sites with water flashing had these issues with similar magnitudes, the number of sites with emissions >26 kg CH_4_ per hour would approximate the one derived from the site-based distribution.

### Methods for liquid unloadings

Regional data from the GHGRP^23^ show that 15% of reporting wells indicate venting from liquid unloadings, with an average of 2.4 venting events per well per year for the wells that unload. For 25,700 wells in the region, the total expected events per year would be 9,300. If we assume duration of 1 h, the average frequency of liquid unloadings events for any given working hour of the year would be 2.1 (see below for discussion of possible underreporting of liquid unloadings activity).

Using a bootstrap method, we sampled (with replacement) from the 4,380 working hours of the year, until we assigned the total events per year. Consequently, we determined the expected frequency of venting events per hour due to liquid unloadings to be 2.1 (95% CI: 0.0–5.0).

In each Monte Carlo run, first we sample a frequency of liquid unloading events from the normal distribution generated as a result of the bootstrapping process. Second, we randomly select the sites that are assigned liquid unloadings events. For the selected sites, we assign 60% of the sites as sites without plunger-lift systems and 40% with plunger-lift systems^23^. Finally, we assign EFs by sampling from the distributions of emissions per event reported by Allen *et al*.^3^ For wells without plunger lifts, the average emissions are 490 kg CH_4_ per hour per event (range: 10.6–2,600 kg CH_4_ per hour per event). For wells with plunger lifts, the average emissions are 130 kg CH_4_ per hour per event (range: 1.1–950 kg CH_4_ per hour per event).

As previously mentioned, we assumed that each liquid unloading event lasts 1 h, and that the measured emission rates persist for the entire event. For non-plunger-lift wells measured by Allen *et al*.^3^, the average duration of liquid unloadings events was 1.4 h (range: 0.2–4.5 h). Similarly, for wells with plunger lifts, the average duration for liquid unloading events was 0.4 h (range: 0.01–2.8 h). If we normalize the emission rates by the actual duration of each event, the EFs would change to 360 kg CH_4_ per hour per event (25–1,800 kg CH_4_ per hour per event) for wells without plunger lifts and 420 kg CH_4_ per hour per event (11–2,800 kg CH_4_ per hour per event) for wells with plunger lifts.

When we apply this change to our model, the average emissions from liquid unloadings would increase from 0.040 kg CH_4_ per hour (95% CI: 0.0–0.18 kg CH_4_ per hour) to 0.050 kg CH_4_ per hour (95% CI: 0.0–0.17 kg CH_4_ per hour). In addition, the expected maximum changes are from 530 kg CH_4_ per hour (0–2,600 kg CH_4_ per hour) to 550 kg CH_4_ per hour (0.0–2,400 kg CH_4_ per hour).

The estimated frequency of unloadings used in our model (2.4 venting events per well per year for the 3,900 wells that unload) is based on regional data from the GHGRP^23^. The unloading frequency data reported by Allen *et al*.^3^, based on an operator survey, suggest quality assurance issues with the liquid unloadings activity data reported to the GHGRP (we note that 2014 GHGRP data quality appears to have improved from previous years but still requires quality assurance). Of most relevance to our analysis is Allen *et al*'s data, indicating that wells with plunger lifts vented at least twice as frequently than what we derived from the GHGRP for the Barnett Shale (Supplementary Table 7). A direct comparison with vent frequencies in Allen *et al*.^3^ is complicated, because their results are aggregated into four large geographic regions. Nevertheless, here we consider results in Allen *et al*.^3^ for the Midcontinent (MC) and Gulf Coast (GC) regions to help bound potential uncertainty in our assumed liquid unloading vent frequency.

We used the results of operator surveys reported in Allen *et al*.^3^ to calculate average events per unloading well of 5.5 and 91 for the GC and MC regions, respectively (Supplementary Table 7). The GC average is about twice as high as the value we derived from the GHGRP for Barnett Shale counties but the MC average (which contains the Barnett Shale) is 35 times higher. However, it is important to note that based on GHGRP data^23^, the MC average in Allen *et al*.^3^ appears to be heavily influenced by wells in the Arkoma basin (averaging 69 events per well, which is driven by the very high event frequency for plunger lift wells of ∼400 events per venting well; Supplementary Table 7). Consequently, it is unlikely to be that the venting frequency for unloading wells in the Barnett will be as high as the regional average from MC wells in Allen *et al*.^3^ For comparison, GHGRP data indicates the basin in the GC or MC regions with the next highest event frequency is the Permian with 6.3 events per well (Supplementary Table 7). We acknowledge that additional uncertainty would be caused by an overall underestimation of the frequency of events in the GHGRP—especially from plunger-lift wells, which vent more frequently on average—when compared with results from Allen *et al*.^3^

One potential explanation for the lower value reported to the GHGRP by Barnett operators compared with the results in Allen *et al*.^3^ is that the frequency of events per well that vents for liquid unloading is correct but that non-plunger wells were misclassified as wells with plunger lifts. In this case, a higher number of wells without plunger lifts (and higher EFs) would affect the magnitude of emissions but not the frequency. This effect does not change our main conclusions, as we already know that the magnitude of liquid unloadings is enough to explain sites emitting >26 kg CH_4_ per hour (although total emissions from liquid unloadings would increase slightly, the added contribution to total production site emissions would be small).

The second possible explanation would be that the classification is correct, but the frequency of events is underreported by Barnett operators. For our model, we averaged data from 19 facilities in the region that reported emissions from well venting for liquid unloadings to the GHGRP. The maximum frequency (liquid unloadings events per year) reported by one of these 19 facilities was 6.6 venting events per well, with 24% of reporting wells venting from liquid unloadings. We use this higher frequency and percent of affected wells to test the effect of underreporting the frequency of events in the GHGRP data.

Applying the higher frequency of 6.6 events per well to the alternative count of unloading wells in the region (6,200, or 24% of 25,700 total wells), the total expected events per year would be 40,600. If we assume duration of 1 h, the average frequency of liquid unloadings events for any given working hour of the year would be 9. This yields a frequency that is ∼4 times higher than the frequency used in our model. However, even with this higher frequency of unloadings, our component-based aggregation would still produce a count of high-emitting sites that is roughly an order of magnitude smaller than what is expected from the site-based estimate. This result constrains the effect of potential underreporting of the frequency of liquid unloadings in the region.

In addition, another assumption that could affect the frequency of liquid unloadings is the number of working hours in which an unloading could occur. If we assume 8 working hours instead of 12, we would see a 50% increase in the frequency of these events—still not enough to explain the number of high-emitting sites from the site-based analysis.

### Methods for dehydrators

Glycol dehydrators (dehydrators) are used to remove water from produced natural gas to prevent corrosion and liquid accumulation in pipelines. Although dehydrators are most often found at midstream facilities, dehydrators are also used at natural gas production sites as shown in reports to the GHGRP^23^ (Supplementary Table 8).

We estimated the number of dehydrators on production sites in the Barnett Region using data in the 2014 GHGRP^23^. Twenty two operators submitted reports for production facilities in basins that included counties in the Barnett region (these facilities comprised ∼16,000 oil and gas wells). Eleven of those operators reported 30 dehydrators. The ratio of reported dehydrators to wells in the Barnett region was 0.002. We scaled this ratio to the entire population of wells (25,700 wells with reported natural gas production) to derive an estimate of 51 dehydrators in the Barnett region. For comparison, Barnett Shale operators reported 45 dehydrators in upstream service in 2009 (18 uncontrolled and 28 controlled) in response to a special inventory (BSEI) request from the TCEQ^36^. Each iteration of the Monte Carlo model randomly assigns these 51 dehydrators to individual sites.

In our Monte Carlo analysis, each dehydrator was assigned an emission rate, in kg per hour, randomly selected from the distribution of reported GHGRP emissions for production segment dehydrators in basins containing Barnett counties (Supplementary Table 9). About half of the Barnett dehydrators in the GHGRP are small (<0.4 MMscf per day). The maximum emissions rate for any Barnett dehydrator (0.50 kg per hour) was reported for a small, uncontrolled unit; only one small dehydrator in the Barnett had emission controls. All but 3 large dehydrators (>0.4 MMscf per day) had emission controls; the throughput of the largest uncontrolled unit was 1.1 MMscf per day. As shown in Table 1, dehydrators contribute < 0.3% of total emissions.

No data were obtained about potential emissions from start-up/shutdown of dehydrators used at natural gas production sites. A permit application for a 25 MMscf per day dehydrator at a gathering compressor station in the Barnett Shale (Targa Greenwood Compressor Station) reported dehydrator blowdown emissions of 2,900 scf per hour (one event per year lasting 1 h)—CH_4_ emissions of ∼40 kg per hour. Such infrequent events at the relatively small number of dehydrators in the Barnett region are unlikely to be sampled during routine surveys and would nevertheless be unlikely to have a significant effect on the distribution of site emissions and the magnitude of total population emissions.

It is not surprising that the emission distribution of Barnett dehydrators underpredicts at the high end relative to the national distribution (Supplementary Table 9), considering the small number of Barnett units and the tendency for emissions from oil and gas equipment to exhibit heavily skewed distributions. We examined in two ways the effect on our results of possible bias due to sampling from a distribution that lacks sites emitting at levels characteristic of the tail of the distribution.

First, we considered what emissions from Barnett dehydrators would be if they all emitted at the national average for all large dehydrators (1.4 kg per hour). This would mean that 50 dehydrators would emit a total of ∼70 kg per hour. Total dehydrator emissions would be ∼10 times larger than predicted by our model, but would represent only ∼3% of total component-based emissions. One of the 50 dehydrators could reasonably be expected to emit at the 97.5th percentile of the national population (10 kg per hour). Such a level of emissions would not explain the root cause of site-level emissions >26 kg per hour.

Second, we used the GRI-GLYCalc, Version 4 (GLYCalc)^47^ model to examine whether the high end of the distribution of national GHGRP dehydrator data was consistent with worst-case emissions expected from routine operation of dehydrators. The GLYCalc model is widely used by industry to estimate emissions for air permitting and emission inventory reporting. A detailed summary of this modeling is provided in Supplementary Note 5; here we include a brief summary of results.

The GLYCalc model predicts maximum emissions from routine operation of an uncontrolled glycol dehydrator are on the order of 1 kg per hour for each MMscf per day of gas throughput (Supplementary Table 10). This level of emissions occurs for systems without controls operating at the highest temperatures and contactor pressures (for example, 110 F and 1,100 psi). For reference, the comparable value for median conditions is 0.29 kg per hour-MMscf per day (case 7a). Emissions could be twice as high or more if glycol circulation rates are not optimized for the actual throughput^48^.

For throughputs typical of the median and 97.5th percentile of large dehydrator throughputs at natural gas production facilities reported to the 2014 GHGRP (2 and 25 MMscf per day, respectively), we estimate maximum emissions from dehydrators to be 4 and 50 kg per hour for the median and 97.5th percentile of GHGRP-reported dehydrator throughputs, respectively. These high-end estimates would account for the highest T and P operating conditions and a glycol circulation rate 2 × greater than optimal. For comparison, of 1953 units >0.4 MMscf per day reported in the 2014 GHGRP with nonzero operating hours, 37 units (2%) had emissions per operating hour >10 kg per hour; 13 units (0.7%) had emissions >60 kg per hour; and 2 units (0.1%) had emissions >80 kg per hour (130 and 620 kg per hour). Some of the highest values may be the result of reporting errors.

Key operating parameters and variables used in our GLYCalc modelling are summarized in Supplementary Table 11 and Supplementary Note 5.

### Data availability

All data sets used in this work were made available as part of the publications referenced and described in the text. Code used for the Monte Carlo simulation is available from the authors upon request.

## Additional information

**How to cite this article:** Zavala-Araiza, D. *et al*. Super-emitters in natural gas infrastructure are caused by abnormal process conditions. *Nat. Commun.* **8,** 14012 doi: 10.1038/ncomms14012 (2017).

**Publisher's note:** Springer Nature remains neutral with regard to jurisdictional claims in published maps and institutional affiliations.

## Supplementary Material

Supplementary Information Supplementary Figures, Supplementary Tables, Supplementary Notes and Supplementary References

## Figures and Tables

**Table 1:** Results of Monte Carlo aggregation of component-based emissions in the Barnett Shale.

**Component**	**Emissions (kg CH**_**4**_ **per hour per site)**	**Percent of total emissions**	**Max. emissions (kg CH**_**4**_ **per hour per site)**	**Number of sites where contribution of individual component is >26** **kg CH**_**4**_ **per hour**
Pneumatic controllers	0.53 (0.52–0.54)	46%	9.5 (7.6–13)	0
Chemical injection pumps	0.18 (0.17–0.18)	15%	6.7 (5.0–9.3)	0
Equipment leaks	0.15 (0.14–0.15)	13%	9.0 (6.8–13)	0
Compressors	0.10 (0.10–0.11)	8.7%	18 (14–22)	0
Water tank flashing	0.084 (0.083–0.085)	7.3%	22 (19–26)	0
Condensate/oil tank flashing (60% control efficiency)	0.073 (0.045–0.11)	6.3%	160 (68–340)	10 (5–17)
Liquid unloadings	0.040 (0–0.18)	3.5%	530 (0–2,600)	2 (0–4)
Dehydrators	3 × 10^−4^ (2 × 10^−4^–3 × 10^−4^)	0.03%	0.44 (0.17–0.50)	0
Total	1.2 (1.1–1.3)	100%	600 (90–2,600)	13 (7–20)

Table shows average emissions per site due to each component, contribution to total emissions and maximum expected emissions from each component (95% CI shown in parentheses). Also shown are the average number of sites where individual components produce emissions greater than the 99th percentile of site-based emissions from Zavala-Araiza *et al*.^21^ (wherein 174 sites emit >26 kg CH_4_ per hour and produce 44% of total emissions).
