# Supplementary material for: Super-emitters in natural gas infrastructure are caused by abnormal process conditions
Source: Nat Commun. 2017 Jan 16;8:14012. doi: 10.1038/ncomms14012 (PMC5241676; doi:10.1038/ncomms14012)
Supplement: Supplementary Information — Supplementary Figures, Supplementary Tables, Supplementary Notes and Supplementary References [file ncomms14012-s1.pdf]

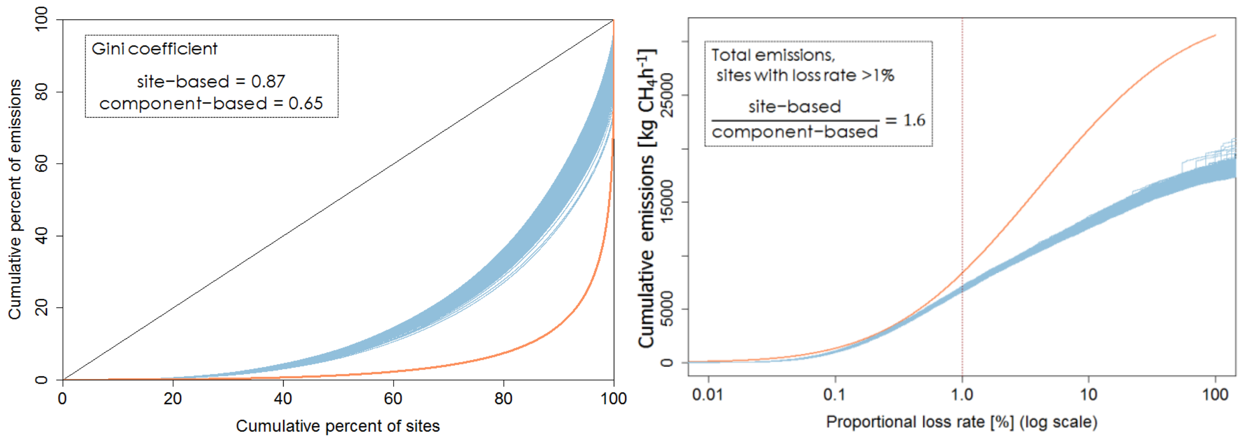

(a) **(b)**

Supplementary Figure 1 | Distribution of methane emissions from production sites in the Barnett Shale. Blue lines represent each of 10^4 Monte Carlo iterations from the component-based aggregation in this work and orange lines represent the site-based results derived from Zavala-Araiza et al.[1]. (a) Lorenz curve: cumulative percent of emissions vs. cumulative percent of sites. (b) Cumulative emissions as a function of proportional loss rates (emissions normalized by production). In (a), as distributions deviate from the black 45 degree line (equality line) there is an increasingly higher level of disproportionality in the contribution from a smaller fraction of sites. Vertical line in (b) represents sites that emit 1% of their production.

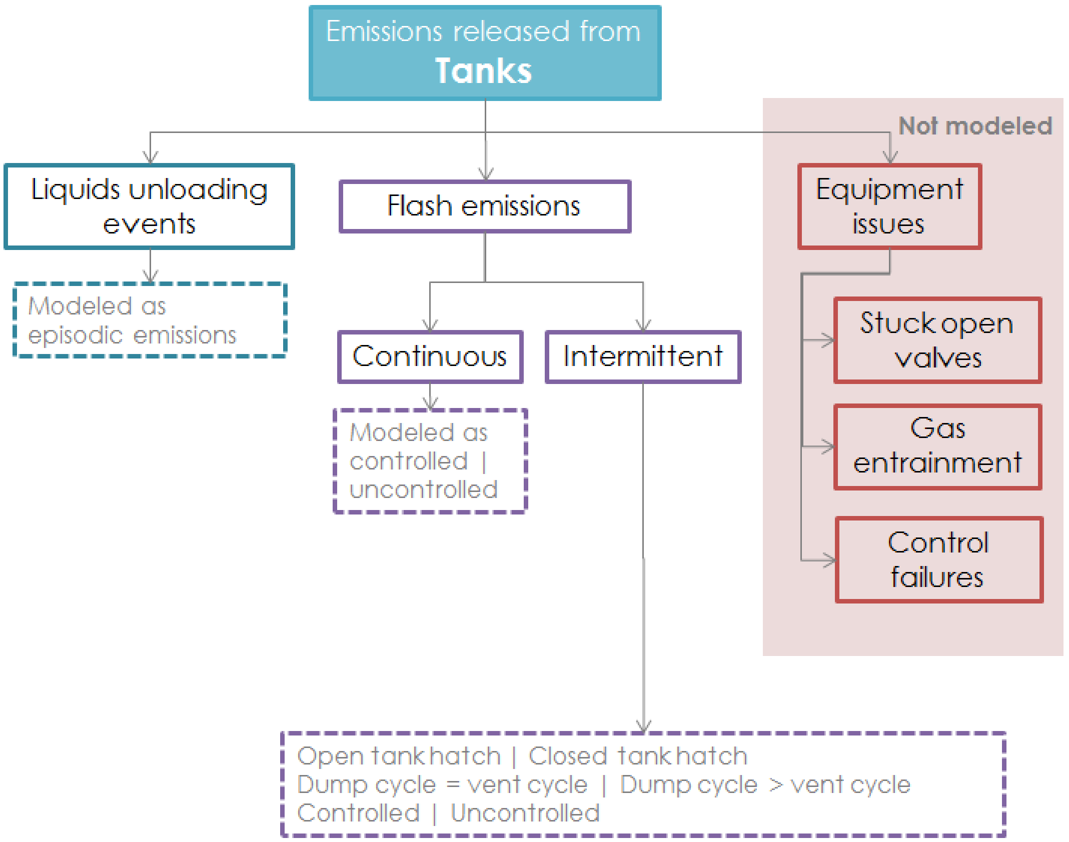

Supplementary Figure 2 | Potential sources of high emissions released through tanks. Flow chart describing possible sources of methane emissions that would be vented to the atmosphere through tanks. Emissions due to equipment issues (or other abnormal conditions) were not modeled in this work because the objective was to determine whether the expected behavior of components explained previously observed site-based emissions. We interpret the significant difference between the component- and site-based estimates to indicate presence of such equipment issues.

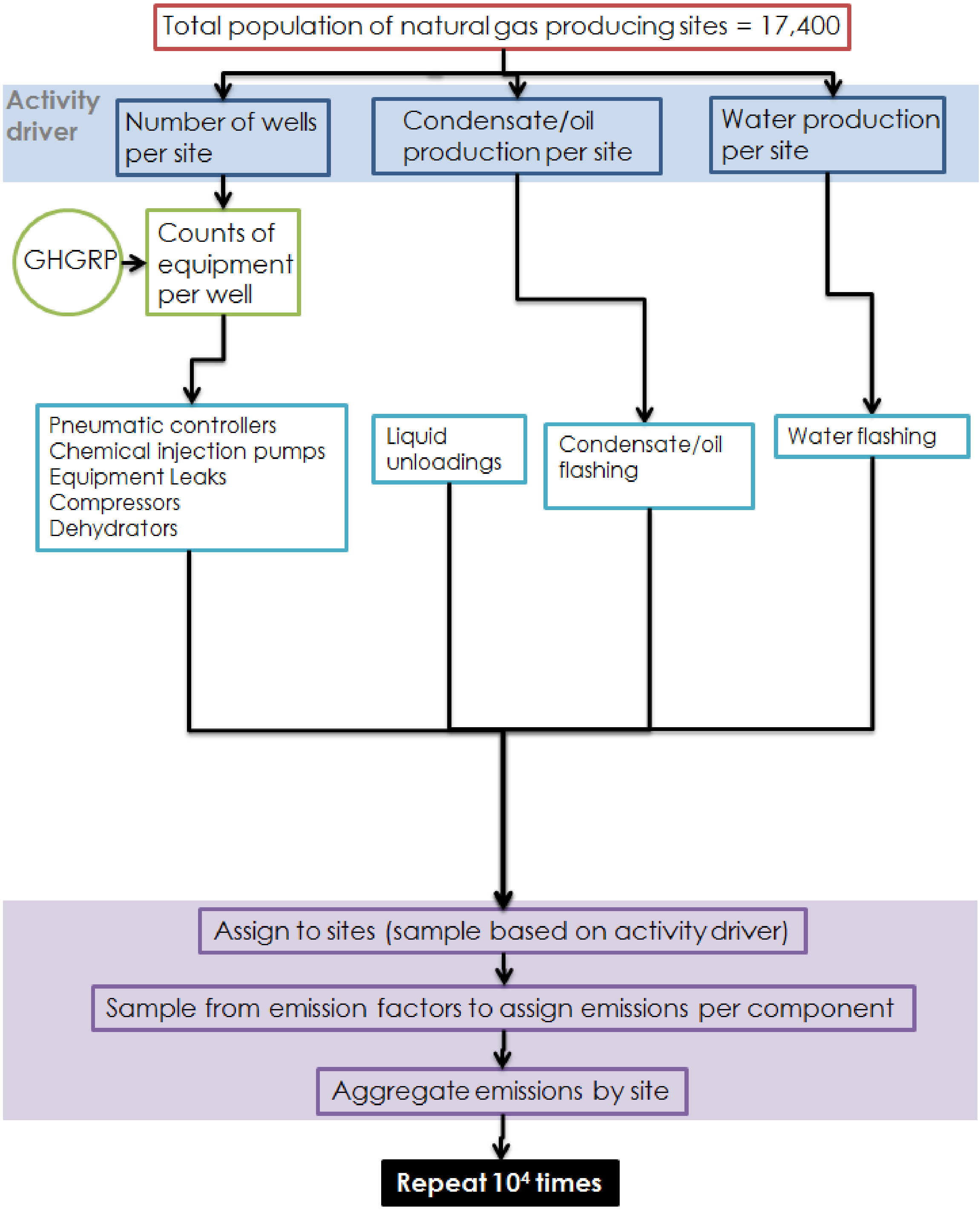

Supplementary Figure 3 | Flow chart summarizing the general methodology used to estimate emissions from every component on natural gas production sites. See Methods for the specific methodologies for each component.

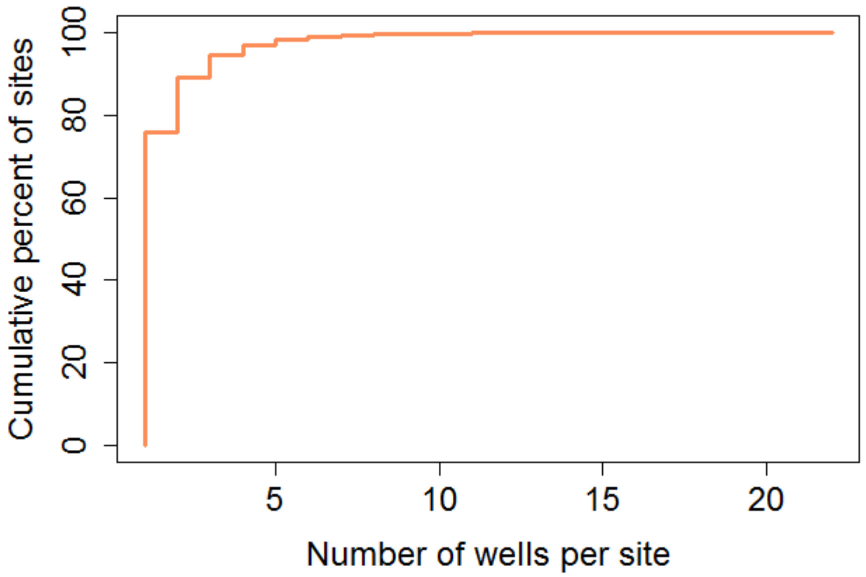

Supplementary Figure 4 | Distribution of number of wells per site for the 17,400 gas producing sites in the Barnett Shale (total number of wells: 25,700).

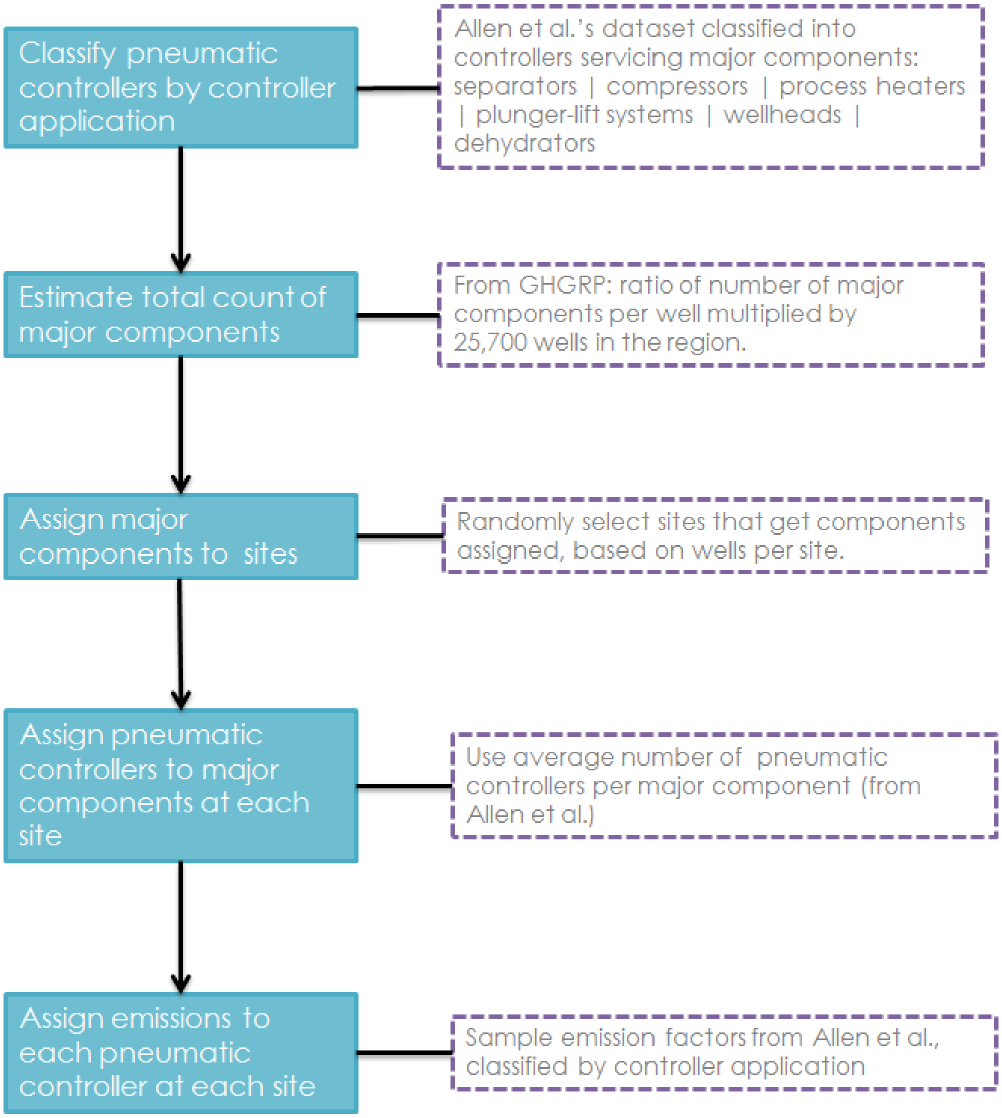

Supplementary Figure 5 | Flow chart summarizing the methodology used to assign pneumatic controllers to sites and estimate their emissions.

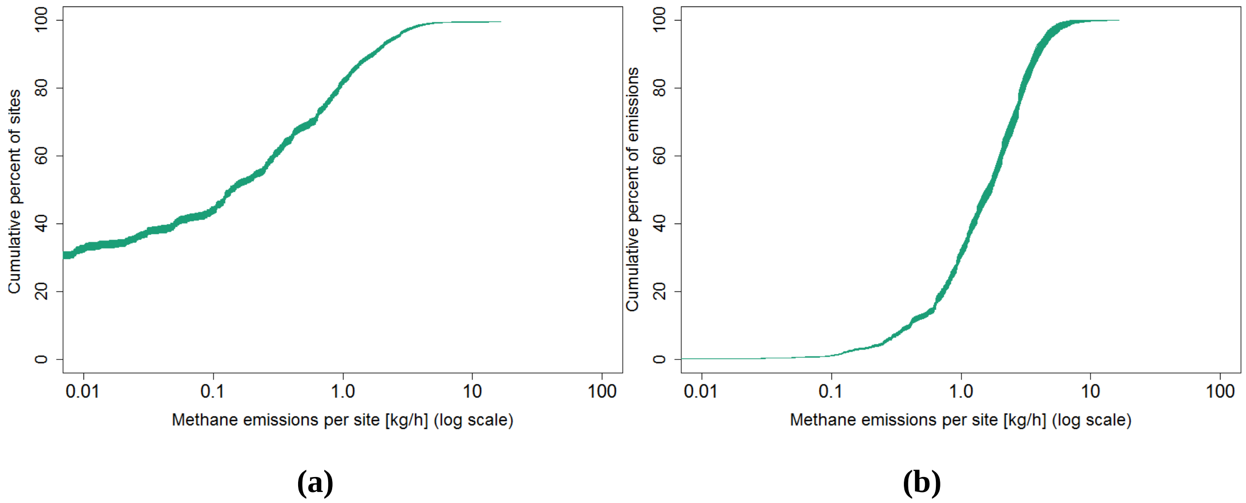

Supplementary Figure 6 | Distribution of emissions from pneumatic controllers per site. Cumulative percent of sites (a) and cumulative percent of emissions (b) as a function of total emissions per site from all controllers.

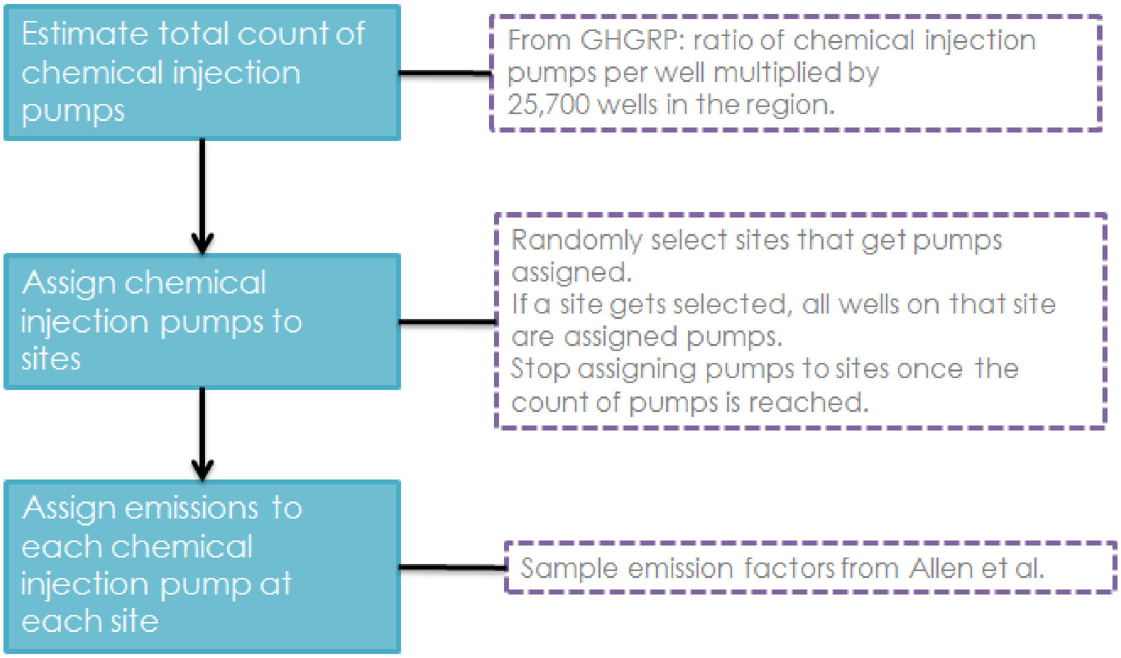

Supplementary Figure 7 | Flow chart summarizing the methodology used to assign chemical injection pumps to sites and estimate their emissions.

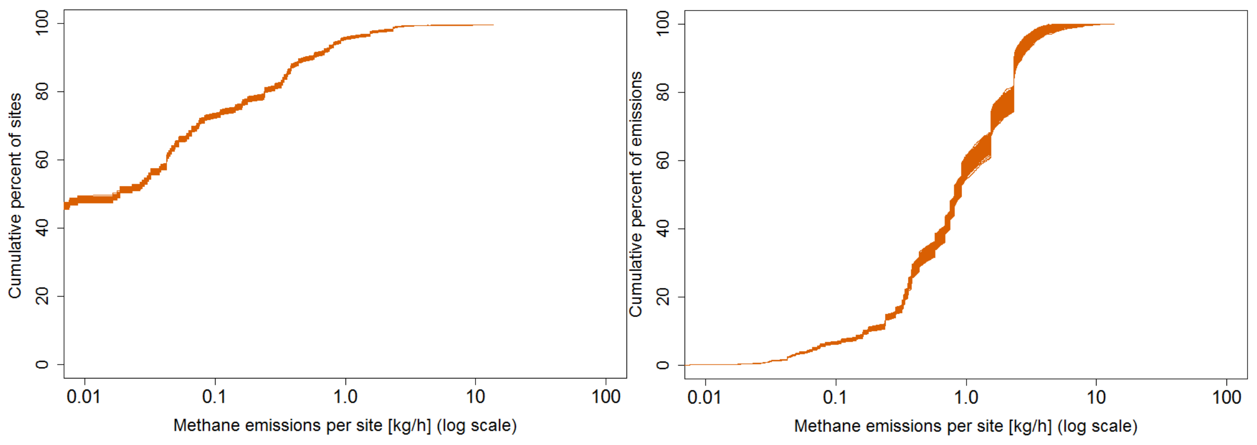

(a) **(b)**

Supplementary Figure 8 | Distribution of emissions from chemical injection pumps per site. Cumulative percent of sites (a) and cumulative percent of emissions (b) as a function of total emissions per site from all pumps.

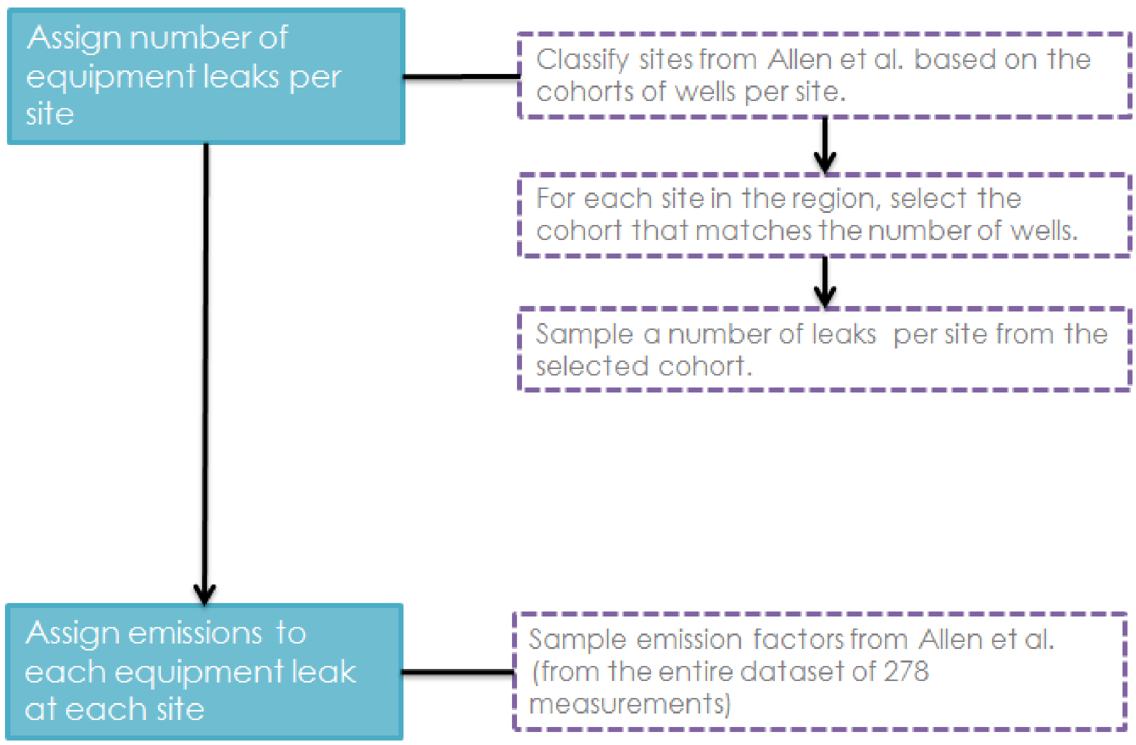

Supplementary Figure 9 | Flow chart summarizing the methodology used to assign equipment leaks to sites and estimate their emissions.

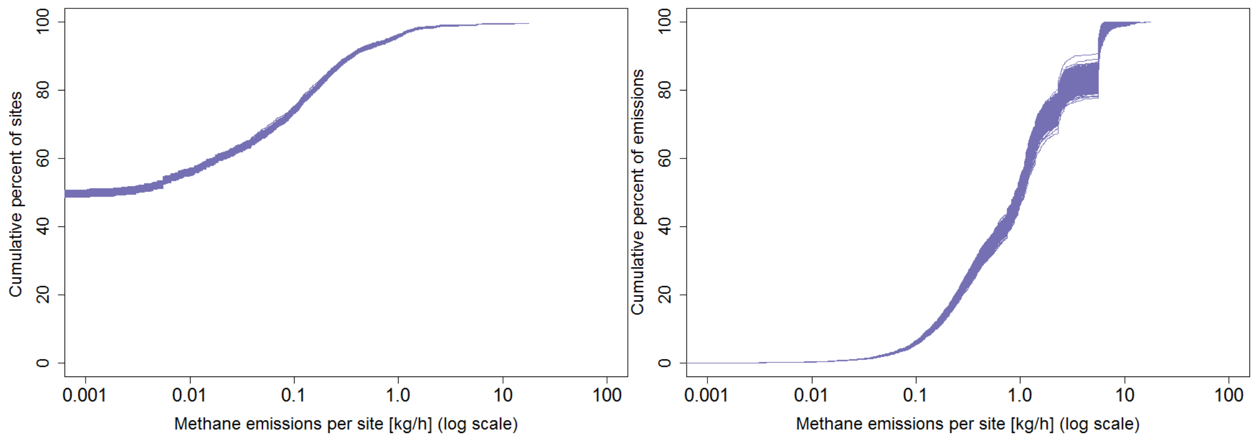

(a) **(b)**

Supplementary Figure 10 | Distribution of emissions from equipment leaks per site. Cumulative percent of sites (a) and cumulative percent of emissions (b) as a function of total emissions per site from all equipment leaks.

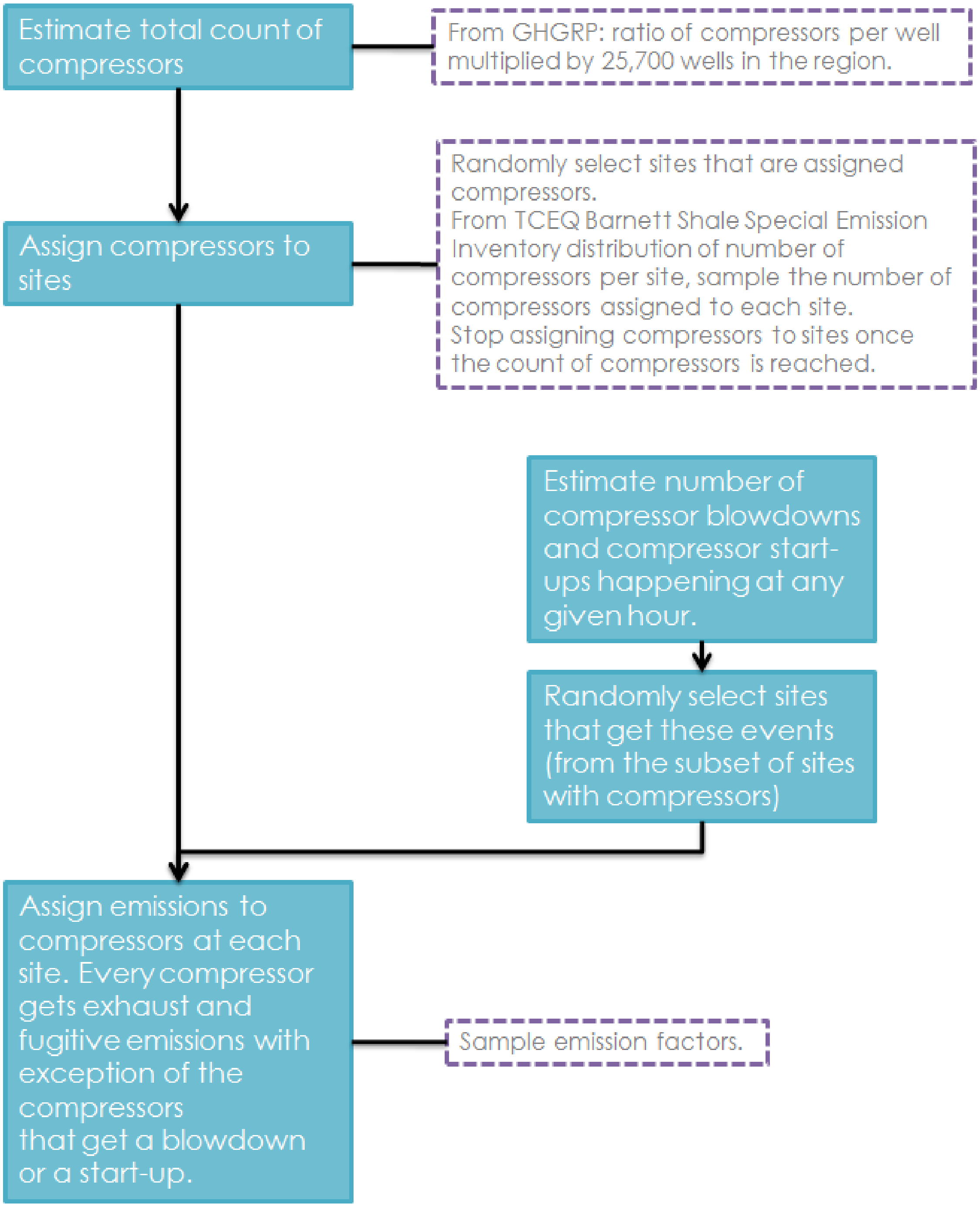

Supplementary Figure 11 | Flow chart summarizing the methodology used to assign compressors to sites and estimate their emissions.

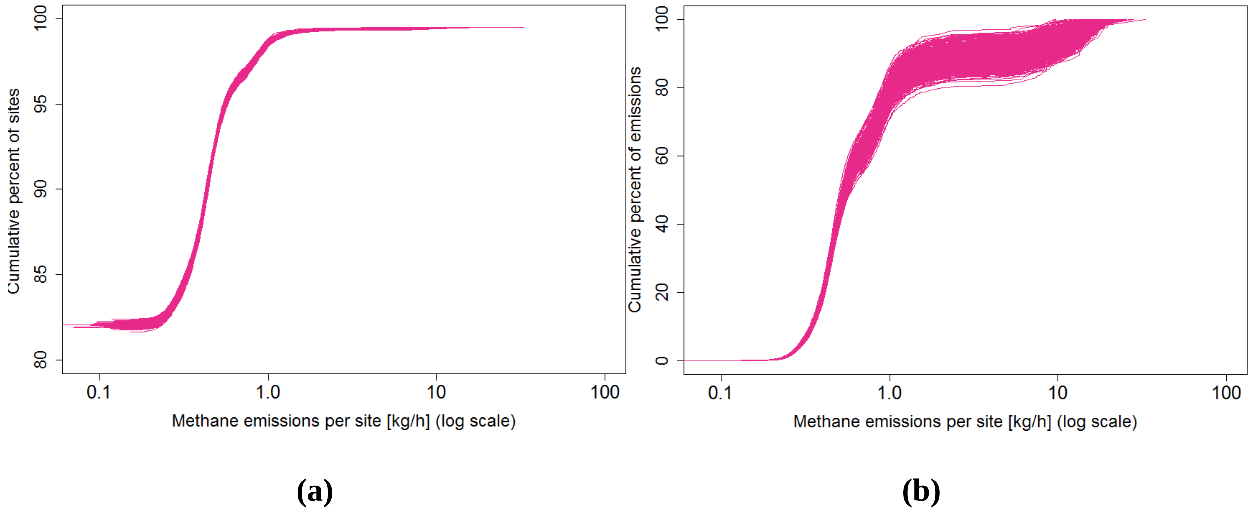

Supplementary Figure 12 | Distribution of emissions from compressors per site. Cumulative percent of sites (a) and cumulative percent of emissions (b) as a function of total emissions per site from all compressors.

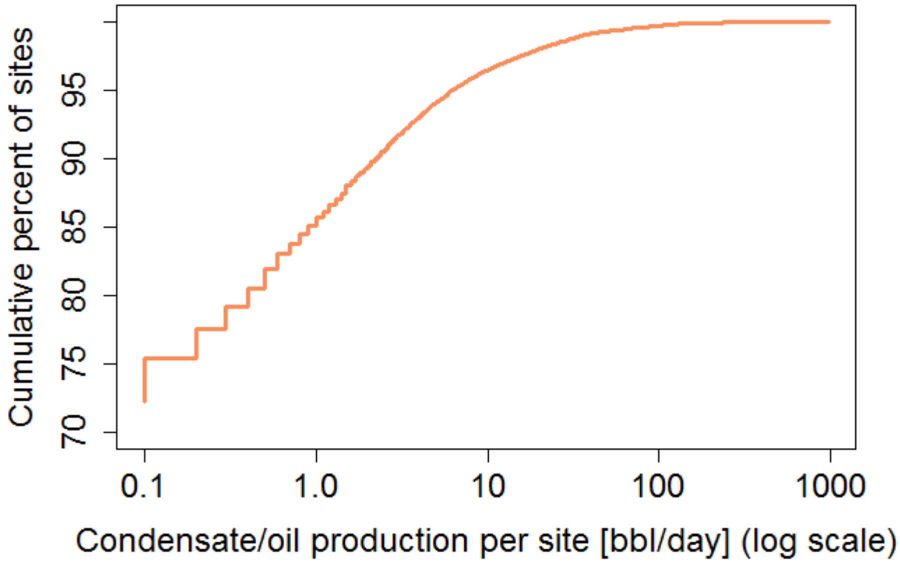

Supplementary Figure 13 | Distribution of condensate/oil production per site. Cumulative percent of sites as a function of oil production rate per site.

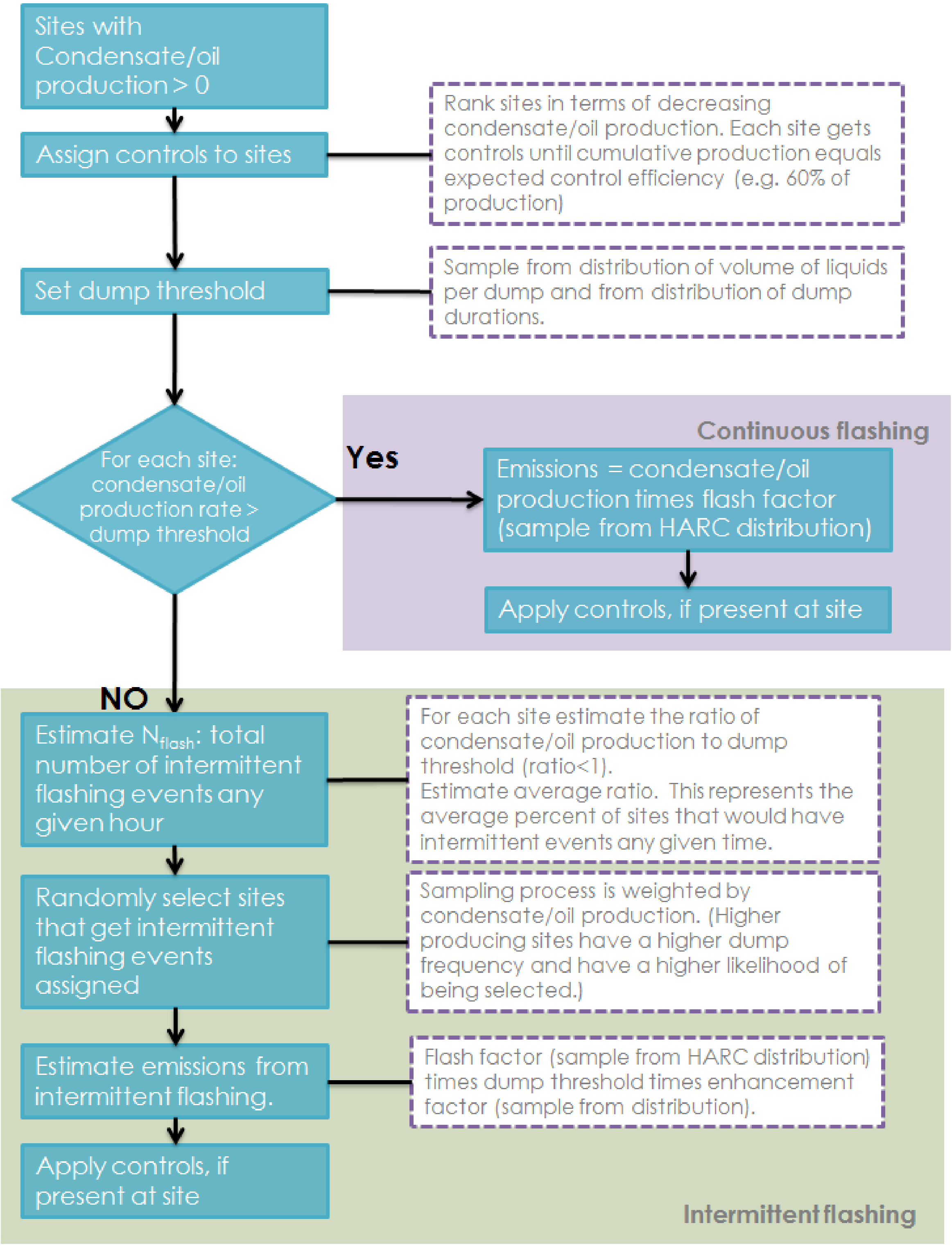

Supplementary Figure 14 | Flow chart summarizing the methodology used to estimate emissions from condensate tank flashing at sites.

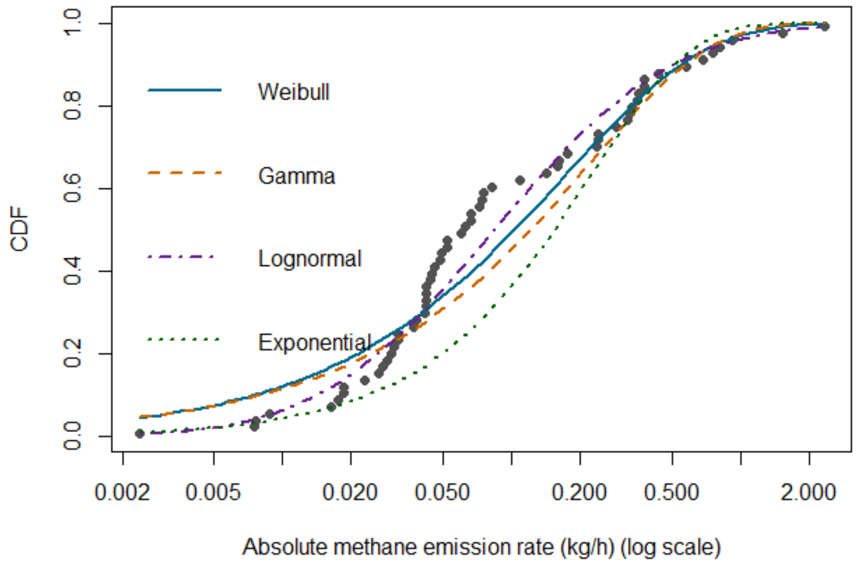

Supplementary Figure 15 | Comparison of the cumulative distribution functions (CDF) for measurements of emissions from chemical injection pumps (See Methods) (dark grey dots), and measurements fitted to a Weibull distribution (blue), gamma distribution (orange), lognormal distribution (purple), and exponential distribution (green).

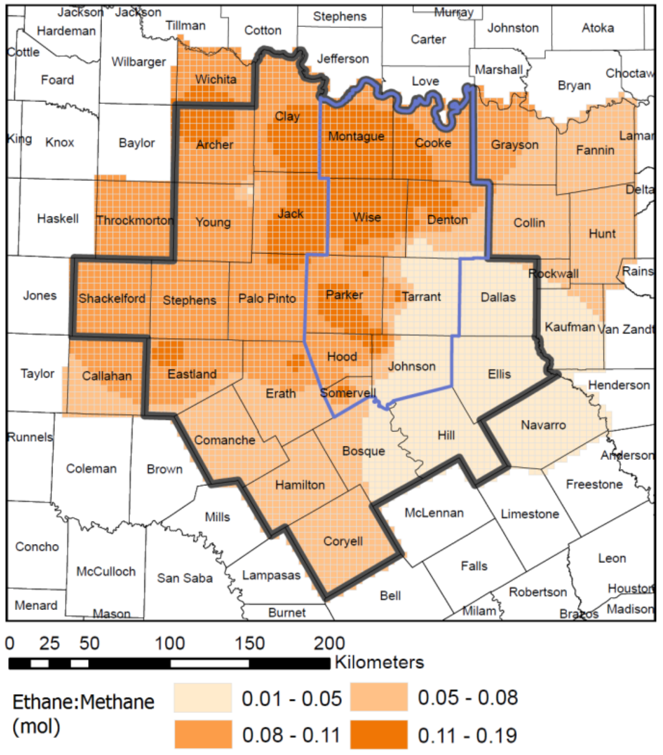

Supplementary Figure 16 | Gridded map showing ethane to methane molar ratios based on interpolated gas composition showing counties in North Central Texas[2]. The darker grid cells represent regions where wet gas is produced. The black margin represents the 25-county Barnett Shale production region and the blue margin represents the counties where measurements were taken.

Supplementary Table 1 | Summary of Barnett Shale activity factors and emission factors for pneumatic controllers, classified by controller application. As a comparison, the EPA Greenhouse Gas Reporting Program (GHGRP)[3] shows a ratio of 2.8 pneumatic controllers per well, here we estimate an average of 2.3 pneumatic controllers per well.

**Application**	**Count of pneumatic controllers per equipment (derived from Allen et al.[4])**	**Count of equipment per well (derived from GHGRP)[3]**	**Number of measured pneumatic controllers* in Allen et al.[4]**	**Emissions per controller in kg CH_4h^{-1}. Arithmetic mean and range (derived from Allen et al.[4])**
**Separator**	2-phase separator : 1<br>3-phase separator: 2	Site with gas production only: 1 2-phase separator<br>Site with oil/condensate and gas production: 1 3-phase separator and 0.06 2-phase separators	186	0.14 (0.0 – 2.8)
**Process heater**	1.5	0.009	45	0.008 (0.0 – 0.315)
**Compressor**	4.3	0.14	20	0.24 (0.0 – 1.7)
**Dehydrator**	2.5	0.002	25	0.045 (0.0 – 0.31)
**Wellhead**	0.42	1	67	0.022 (0.0 – 0.21)
**Plunger-lift**	0.90	40% of the wells with liquids unloadings that vent have plunger-lifts	30	0.072 (0.0 – 0.92)

*Three measured devices had 'Flare' as application and 1 device had 'Sales' as application. All of these four devices had an emission rate of 0.0 kg CH_4h^{-1}. They were not considered in the analysis because of the limited number of measurements.

Supplementary Table 2 | Sites where Allen et al.[5] measured equipment leaks were classified into cohorts based on the number of wells per site. For each cohort, the table summarizes the number of sampled sites and the distribution of leaks per site.

**Number of wells per site**	**Number of sampled sites**	**Mean leak count per site (range)**
1	51	1 (0 – 5)
2	16	2 (0 – 9)
3	25	2 (0 - 10)
4	10	2 (0 – 5)
5	17	3 (0 – 9)
6	14	4 (0 - 12)
Equal to or greater than 7*	13	3 (0 – 8)

*Sites with 7 or more wells were grouped into a single cohort because of the small sample size. The maximum number of wells at a sampled site was 16.

Supplementary Table 3 | Comparison of the HARC[6] dataset used in this study to estimate flash emissions in the Barnett Shale to other datasets.

Study	N	API Gravity	Separator pressure (psig)	Condensate/oil production (bbl/ d^{-1})	Measured vent gas (scf bbl^{-1})	CH_4-specific emission rate (scf bbl^{-1})
**HARC[6]**	12*	55 (41 - 61)	36 - ~200	26 (1 - 180)**	226 (50 - 686)	76 (17 - 412)
**Hy-Bon[7]**	6	61(55 - 71)	125 – 231	22 (5 - 62)	240 (47 - 592)	66 (5 - 184)
**Environ[8]**	3	58 (51 - 61)	119 – 171	21 (1 - 59)	181 (125 - 253)***	48 (40 - 74)

* Includes 2 oil tanks; excludes 3 tanks flagged for possible contribution of measurement error or non-flash emissions.

** Average of condensate tanks was 5 bbl d^{-1}

*** Averages of two different methods.

Supplementary Table 4 | Measured Tank emissions data for Barnett Shale Production Sites as reported to the EPA Greenhouse Gas Reporting Program (GHGRP)[3]. (Based on 21 sub-basin/company records using tank Reporting Methods 1 & 2.

Method	Average separator temperature (°F)	Average separator pressure (psig)	API Gravity (degrees)	Minimum flash gas CH_4 (volumetric fraction)	Maximum flash gas CH_4 (volumetric fraction)	CH_4-specific emission rate (scf bbl^{-1})*
**Mean**	80	149	47	0.23	0.31	317
**Oil production weighted average**	78	127	44	0.17	0.34	269
**Minimum**	60	25	38	0.14	0.01	2
**Median**	84	105	43	0.22	0.25	71
**Maximum**	108	377	61	0.40	0.63	1625

* The scf bbl^{-1} is a potential emission factor that includes flared CH_4 back calculated from CO_2 emissions.

Supplementary Table 5 | Emissions from condensate tank flashing under different assumptions of controls.

Control efficiency	Emissions per site from condensate tank flashing only (kg CH_4 h^{-1})	Maximum emissions per site from condensate tank flashing only (kg CH_4 h^{-1})	Total emissions per site – all components	Sites with condensate tank flashing emissions >2.7 kg CH_4 h^{-1}	Sites with condensate tank flashing emissions >26 kg CH_4 h^{-1}
20% control efficiency	0.13<br>(0.10 – 0.17)	190<br>(88 - 360)	1.2<br>(1.2 – 1.3)	120<br>(61 - 160)	19<br>(12 - 29)
60% control efficiency (main assumption)	0.073<br>(0.045 – 0.11)	160<br>(68 - 340)	1.2<br>(1.10 – 1.3)	64<br>(31 - 93)	10<br>(5 - 17)
75% control efficiency	0.048<br>(0.027 – 0.077)	140<br>(47 - 310)	1.1<br>(1.1 – 1.3)	42<br>(22 - 61)	7<br>(2 - 12)
90% control efficiency	0.021<br>(0.008 – 0.039)	100<br>(20 - 260)	1.1<br>(1.1 – 1.2)	17<br>(8 - 28)	3<br>(0 - 6)

Supplementary Table 6 | Summary of controlled sites under different assumptions of controls.

Control efficiency	Minimum condensate/oil production from a controlled site (bbl d^{-1})	Number of controlled sites (percent of total sites with oil/condensate production)	Number of total wells from controlled sites
20% control efficiency	150	20 (0.5%)	62
60% control efficiency (main assumption)	27	250 (5%)	630
75% control efficiency	12	530 (12%)	1,200
90% control efficiency	3.5	1,300 (30%)	2,600

Supplementary Table 7 | Summary of 2014 EPA Greenhouse Gas Reporting Program (GHGRP) data[3] for liquid unloadings for basins corresponding to the Midcontinent and Gulf Coast regions in Allen et al.[9] The last three rows summarize the aggregation of data for Barnett Shale counties as used in this work and the operator survey data reported in Allen et al.[9]

				no plunger lift			plunger lift			total		
Region in Allen et al.	Basin ID	Basin	reporting facilities	unloading wells that vent	events	events per venting well	unloading wells that vent	events	events per venting well	unloading wells that vent	events	events per venting well
MC	400*	Ouachita Folded Belt	1	0	0	NA	1	1	1.0	1	1	1.0
MC	415*	Strawn Basin	7	386	799	2.1	558	2,303	4.1	944	3,102	3.3
MC	420*	Fort Worth Syncline	10	352	634	1.8	915	1,990	2.2	1,267	2,624	2.1
MC	350**	South Oklahoma Folded Belt	7	33	45	1.4	53	100	1.9	86	145	1.7
MC	345	Arkoma Basin	12	2,901	32,775	11.3	505	203,435	403	3,406	236,210	69.4
MC	355	Chautauqua Platform	3	83	232	2.8	29	106	3.7	112	338	3.0
MC	360	Anadarko Basin	24	501	1,921	3.8	816	5,332	6.5	1,317	7,253	5.5
MC	430	Permian Basin	19	805	2,456	3.1	544	6,041	11.1	1,349	8,497	6.3
GC	210	Mid-Gulf Coast Basin	1	5	5	1.0	0	0	NA	5	5	1.0
GC	220	Gulf Coast Basin (LA, TX)	26	2,637	5,912	2.2	167	967	5.8	2,804	6,879	2.5
GC	230	Arkla Basin	15	690	3,025	4.4	213	791	3.7	903	3,816	4.2
GC	260	East Texas Basin	17	1,561	8,977	5.8	477	1,956	4.1	2,038	10,933	5.4
MC	N/A	GHGRP data for "Barnett Counties" (this work)	19	739	1,434	1.9	1,489	4,313	2.9	2,228	5,747	2.6
MC	N/A	Allen et al.[24] Regionwide average (company survey)	N/A	2,824	22,100	7.8	1,048	328,760	314	3,872	350,860	90.6
GC	N/A	Allen et al.[24] Regionwide average (company survey)	N/A	1,712	9,216	5.4	426	2,603	6.1	2,138	11,819	5.5

* fully included in average for Barnett counties
** only one facility had operations in Barnett counties

Supplementary Table 8 | National 2014 EPA Greenhouse Gas Reporting Program (GHGRP)[3] data on dehydrators in the oil and gas production segment.

Throughput	N	Total reported emissions (MT CH_4)	% of reported emissions	% of units with reported emission controls
>4 MMscf d^{-1}	1,956	27,200	78%	54%
<4 MMscf d^{-1}	5,761	7,500	22%	9%
All dehydrators	7,717	34,600	100%	21%

Supplementary Table 9 | Statistical summary of reported emissions and operating characteristics for ~2,000 large dehydrators (>0.4 MMscfd) in all U.S. basins (left) and 30 dehydrators in the Barnett Shale (right)[3].

	All Basins (dehys > 0.4 MMscfd); N ≈ 2,000					Barnett only (all dehys); N ≈ 30				
Statistical Metric	Reported emissions (kg CH4 hr-1)	Operation (hrs)	Throughput (MMscfd)	P (psia)	T (°F)	Reported emissions (kg CH4 hr-1)	Operation (hrs)	Throughput (MMscfd)	P (psia)	T (°F)
Average	1.4	8,108	6	594	78	0.11	8,483	1.9	749	87
2.5%	0	1,512	0.41	132	60	0.0062	7,796	<0.4	213	72
25%	0.019	8,760	0.77	316	70	0.0078	8,483	<0.4	786	89
Median	0.053	8,760	1.7	546	75	0.17	8,483	<0.4	840	90
75%	0.48	8,760	6.5	861	85	0.17	8,760	1.4	840	90
97.5%	10	8,760	25	1,148	110	0.24	8,760	11	940	90

Notes: Reported data was filtered to exclude entries with unrealistic values such as zero for P or T; percentiles are reported independently for each parameter; because dehydrators below 0.4 MMscf d^{-1} do not report P and T, the Barnett percentiles for P and T are for units >0.4 MMscf d^{-1}.

Supplementary Table 10 | GRI-GLYCalc Ver. 4.0 simulation Runs used in this work (1 MMscf d^{-1} Dehydrators without flash tanks, at a variety of pressures, temperatures and gas composition).

	Inputs						Calculated CH_4 emissions	
**Case**	Wet Gas Methane	Wet Gas Throughput (MMSCFD)	Contactor Pressure (psig)	Contactor Temp (F)	Dry Gas (lbs/MMSCF)	Lean Glycol Circulation Rate (gpm)	Calculated Still Column CH_4 (scf h^{-1})	Calculated Still Column CH_4 (kg h^{-1})
**7a**	90%	1.0	600	75	7	0.07	15.1	0.29
**7b**	90%	1.0	600	110	7	0.23	45.4	0.87
**7c**	90%	1.0	600	60	7	0.04	8.2	0.16
**8a**	90%	1.0	1100	75	7	0.04	16.8	0.32
**8b**	90%	1.0	1100	110	7	0.14	52.4	1.01
**8c**	90%	1.0	1100	60	7	0.02	8.4	0.16
**9a**	75%	1.0	600	75	7	0.07	13.7	0.26
**9b**	75%	1.0	600	110	7	0.23	40.4	0.77
**9c**	75%	1.0	600	60	7	0.04	7.6	0.15
**10a**	75%	1.0	1100	75	7	0.04	16.7	0.32
**10b**	75%	1.0	1100	110	7	0.14	49.4	0.95
**10c**	75%	1.0	1100	60	7	0.02	8.7	0.17
**11a**	90%	1.0	200	75	7	0.20	14.1	0.27
**11b**	90%	1.0	200	110	7	0.60	40.4	0.77
**11c**	90%	1.0	200	60	7	0.11	8.3	0.16
**12a**	75%	1.0	200	75	7	0.20	12.1	0.23
**12b**	75%	1.0	200	110	7	0.60	34.4	0.66
**12c**	75%	1.0	200	60	7	0.11	7.1	0.14

Supplementary Table 11 | Modeling Parameters for Cases 7 to 12 - no flash tank and dry gas at 7 lb $MMscf^{-1}$.

**Glycol type:**	TEG	
**Natural gas throughput**	1	MMscf d^{-1}
**Inlet wet gas water content:**	Saturated based on inlet gas press. & temp.	
**Dry gas water content:**	7	lbs water per MMscf
**Lean glycol circulation rate:**	3	gallons of TEG per lb of water removed
**Lean glycol water content:**	1	percent by weight
**Glycol pump type:**	Gas Injection	
**CH4 content of wet gas:**	75%, 90%	by volume
**Inlet wet gas pressures:**	600, 200, 1100	psig - median, 5th, and 95th percentile GHGRP parameters
**Inlet wet gas temperatures:**	75, 60, 110	°F - median, 5th, and 95th percentile GHGRP parameters
**Use of a flash tank:**	None	
**Flash tank pressures:**	N/A	
**Flash tank temperatures:**	N/A	
**Use of stripping gas:**	None	

Supplementary Table 12 | Number of wells and sites from the counties where measurements were taken to create the datasets used in Zavala-Araiza et al.[1] to generate the site-based emission distribution to which we compare our component-based distribution.

County	Total number of sites	Total number of wells	Number of measured sites
**Cooke**	220	420	5
**Denton**	2,000	2,900	110
**Hood**	430	750	1
**Johnson**	1,500	3,100	49
**Montague**	600	1,100	5
**Parker**	1,200	1,700	8
**Somervell**	38	79	1
**Tarrant**	1,300	3,800	31
**Wise**	3,800	4,600	64
**Total**	**11,000**	**18,000**	**270**
**Percent of Barnett Shale total**	**63%**	**71%**	**-**

Supplementary Note 1 | Additional analysis comparing our component-based aggregation of emissions to the site-based estimate

As described in the main text, the component-based emission distribution is less skewed than the site-based distribution. This difference also points to the existence of high unintended emissions. Supplementary Figure 1a shows the graphical representation of the Gini coefficient and the different disproportionalities in the distribution of emissions. As the lines in the Lorenz curves get farther away from the 45 degree line, there is a higher disproportionate contribution form an ever smaller subset of sites[10]. Similarly, as the Gini coefficient of a distribution increases across its range (0-1), there is a more disproportionate contribution to emissions from an ever smaller subset of sites. [10] The Gini coefficient of the site-based distribution is 0.87, while the component-based aggregation generates an average Gini coefficient of 0.65 (95% CI: 0.63-0.68).

The differing Gini coefficients point to a less disproportionate contribution of high-emitting sites to total emissions in the component-based aggregation. If the modeled component-based behaviors do not generate enough high-emitting sites, a likely explanation for the discrepancy between the component-based and site-based estimates is the existence of super-emitting sites, where unintended pathways cause substantial additional emissions of produced gas.

The component-based analysis in this work predicts that 47% of sites would have loss rates >1%, somewhat higher than the site-based distribution. However, cumulative emissions from sites with component-based loss rates >1% are only 13,000 kg CH_4 h^{-1} (95% CI: 12,000 – 16,000 kg CH_4 h^{-1}), significantly lower than for the site-based distribution. (Supplementary Figure 1b). The larger fraction of sites with component-based loss rates >1% is due mainly to our model's over-prediction of emissions for the lowest emitting 90% of sites (Figure 2a, main text).

Underestimation of high-emitters with high proportional loss rates supports the existence of abnormal process conditions that result in emissions which have been empirically observed through site-level measurements but which are not factored into the component-based aggregation that considers only routine behavior.

Supplementary Note 2 | Plausibility of emission rates > the highest reported measurement

The site-based emission distribution includes 5 sites out of 17,400 that would emit more than the highest reported measurement of ~300 kg CH_4 h^{-1} [1]. Such emissions are plausible considering the number of high-production Barnett sites and the reported range of loss rates for such sites.

For example, an instantaneous site-wide loss rate of 5% at the highest gas-producing site would produce 1,000 kg CH_4 h^{-1}, close to the maximum of the site-level distribution. If a single well at this 7-well site had an instantaneous loss rate of 30%, emissions would be ~870 kg CH_4 h^{-1}. More generally, the 5 sites emitting >300 kg CH_4 h^{-1} in the site distribution would correspond to ~3% of sites with high enough production exhibiting site-wide instantaneous loss rates of 10%.

This fraction of sites is similar to the 4% super-emitter probability reported for transmission compressor stations[11]. This analysis assumes that emissions at each production site are constrained by the total volume of gas produced at each site. We acknowledge that instantaneous emissions rates (emission episodes resulting from tanks, liquid unloadings, or abnormal process conditions) may exceed average production rates. Under these circumstances, the fraction of sites that could potentially emit above the reported measurements is even higher, increasing the plausibility of sites having the emission rates predicted by the statistical estimator.

Additional empirical evidence of the fraction of high-emitting sites is provided by an aerial infrared camera survey of over 8,000 natural gas producing sites in seven U.S. regions, including the Barnett.[12] That study found that 3.5% of sites in the Barnett had individual hydrocarbon sources with emissions >3-10 kg h^{-1}. This fraction of sites is similar to the one that would be predicted from the pdf derived by Zavala-Araiza et al.[1], where 9.2% and 3.2% would have total site-wide emissions above 3 kg CH_4/h and 10 kg CH_4 h^{-1}, respectively.

Supplementary Note 3 | Analysis of skewed distributions for individual components

As described in Methods, for components where we had measurements (e.g., pneumatic controllers, chemical injection pumps, equipment leaks), we sample from the distribution of measurements as we assign emissions for each component at each site during the Monte Carlo aggregation routine. The component measurement distributions from which we sample are skewed (e.g., 13% of the measured pneumatic controllers account for 88% of the emissions, 10% of the chemical injection pumps account for 50% of the emissions, 11% of equipment leaks accounted for 70% of emissions), hence our model partially captures the effect of components with fat tails and unintended emissions. In the case of pneumatic controllers a subset of the measurements were clearly identified as devices with equipment issues[4]. We acknowledge that part of the uncertainty in our methods includes the possibility of higher emissions from these component types that are not captured by the sampled datasets, although based on the following analysis we believe any effect would likely be small.

As an exploration of the effect of high emitters above the maximum measured emission rate, we fit measurements of chemical injection pumps to different probability distribution functions and show that they do not affect the conclusions reached in our study. We selected chemical injection pumps based on the fact that it represents the most homogeneous component-level distribution- compared to pneumatics or equipment leaks where the sampled distribution represents a variety of devices and subsets.

We fit the measurements described in Methods to four different probability distribution functions that are expected to characterize the skewedness of the measured data: Gamma, Lognormal,

Weibull and Exponential. We estimated the parameters of each distribution via maximum likelihood estimation (MLE), Supplementary Figure 15 compares the cumulative distribution function for the measurements and the different fits.

We selected the lognormal distribution based on the lower Akaike information criterion (AIC) with parameters μ = -2.5, σ = 1.4. We use the Kolmogorov-Smirnov test to assess the goodness of fit of the lognormal distribution. For a p-value of 0.28 (significance level = 0.05) we accept the null hypothesis stating that the sampled data follows the proposed lognormal distribution.

Once we run our Monte Carlo aggregation routine with the fitted distribution, average emissions per site for chemical injection pumps stay the same 0.18 kg CH_4 h^{-1} (CI: 0.17 – 0.19) but the maximum emissions shift from 6.7 (CI: 5.0 – 9.3) (See Table 1, main text) to 23 (CI: 11 - 58). This change in maximum emissions doesn't significantly change the number of sites where the contribution of chemical injection pumps is >26 kg CH_4 h^{-1}- still at 0 (CI: 0 - 2).

Supplementary Note 4 | Additional background on site-based emission estimates

Zavala-Araiza et al.[1] used a statistical estimator to produce an integrated emissions probability density function (pdf) from systematic and high emitter-biased samples. This pdf was then used to derive facility-level emission factors. A similar method was used for compressor stations, processing plants, and production sites; here we focus on the latter.

Supplementary Figure 16 shows the ethane to methane molar ratio (C2:C1) of produced gas in the Barnett Shale region. To produce this gridded map, 173 data points of gas composition were used[13-15]. Assuming that gas composition is well characterized by the spatial location of sites, it is expected that the missing grid cells can be estimated from neighboring grid cells with known gas composition using an inverse distance weight (IDW) method for interpolation.

Supplementary Table 12 summarizes the sample size and total population of wells and sites in the Barnett Shale counties where measurements were taken. It is clear that the Barnett is a significantly heterogeneous region in terms of gas composition, however, the counties where measurements were taken largely captures this heterogeneity.

Supplementary Note 5 | Parameters and variables used in our GLYCalc modeling

Glycol circulation rate in gallons per minute. The TEG circulation rate for a set of operating parameters is calculated by GLYCalc based on 3 gallons of TEG per lb of water removed. There is a linear relationship between glycol circulation rate and CH_4 emissions when other input parameters are held constant.

Inlet wet gas water content. Assumed to be saturated based on temperature and pressure of the inlet wet gas.

Dry gas water content. Modeling runs include dry gas inputs of 7 lbs water per MMscf and 3 lbs water per MMscf.

Use of a flash tank (gas-condensate-glycol separator or glycol separator). If a flash tank is not used, then the flash gas (from entrained gas from the contact tower) will be liberated in the reboiler and the flash gas will be vented via the regenerator (still column vent). Modeling runs evaluated emissions with and without use of a flash tank, but we only report emissions without the flash tank.

Assumed no controls for regenerator (still column vent).

Contactor pressure. Based on GHGRP data, we modeled a central estimate of 600 psi, with 200 psi and 1100 psi used to estimate low and high-end contactor pressures.

No stripping gas modeled.

Emissions from pneumatic controllers and fugitive leaks associated with a dehydration system are not included in the GLYCalc model (treated under the specific component types).

Supplementary References:

1. Zavala-Araiza, D. et al. Reconciling divergent estimates of oil and gas methane emissions. *Proc. Natl. Acad. Sci. U.S A.* **112,** 15597−15602 (2015).

2. Political boundaries shapefile for Texas. Available at https://tnris.org/data-catalog/entry/political-boundaries/. (Accessed: November 11, 2016).

3. US EPA. Greenhouse Gas Reporting Program. Available at: http://ghgdata.epa.gov/ghgp/main.do. (Accessed: October 14, 2015).

4. Allen, D. T. et al. Methane emissions from process equipment at natural gas production sites in the United States: pneumatic controllers. *Environ. Sci. Technol.* **49,** 633−640 (2015).

5. Allen, D. T. et al. Measurements of methane emissions at natural gas production sites in the United States. *Proc. Natl. Acad. Sci. U.S.A.* **110,** 17768−73 (2013).

6. Hendler, A., Nunn, J., Lundeen, J., McKaskle, R. VOC emissions from oil and condensate storage tanks. Final Report (2009). Available at:

http://files.harc.edu/projects/airquality/projects/H051C/H051Cfinalreport.pdf (accessed February 2013).

7. Gidney, B., Pena, S. Upstream Oil and Gas Storage Tank Project Flash Emissions Models Evaluation Final Report. 2009. Available at: https://www.tceq.texas.gov/assets/public/implementation/air/am/contracts/reports/ei/20090716-ergi-UpstreamOilGasTankEIModels.pdf

8. ENVIRON International. Upstream Oil and Gas Tank Emission Measurements TCEQ Project 2010 – 39. 2010. Available at: https://www.tceq.texas.gov/assets/public/implementation/air/am/contracts/reports/ei/5820784004FY1025-20100830-environ-Oil_Gas_Tank_Emission_Measurements.pdf

9. Allen, D. T. et al. Methane emissions from process equipment at natural gas production sites in the United States: liquid unloadings. *Environ. Sci. Technol*. **49,** 641−648 (2015).

10. Collins M.B., Munoz I., JaJa J. Linking “toxic outliers” to environmental justice communities. *Environ Res Lett.* **11,** 15004 (2016).

11. Zimmerle D.J. et al. Methane emissions from the natural gas transmission and storage system in the United States. *Environ Sci Technol.* **49,** 9374–83 (2015).

12. Lyon D.R. et al. Aerial surveys of elevated hydrocarbon emissions from oil and gas production sites. *Environ Sci Technol*. **50,** 4877-86 (2016).

13. Texas Commission on Environmental Quality. Barnett Shale Special Inventory, Phase Two Workbook (Excel). Available at: http://www.tceq.texas.gov/assets/public/implementation/air/ie/pseiforms/bshaleworkbook.xls. (accessed: 9th March 2016).

14. Hill, R. J., Jarvie, D. M., Zumberge, J., Henry, M., Pollastro, R. M. Oil and gas geochemistry and petroleum systems of the Fort Worth Basin. *AAPG Bull*. **91,** 445–473 (2007).

15. Zumberge, J., Ferworn, K., Brown, S. Isotopic reversal (‘rollover’) in shale gases produced from the Mississippian Barnett and Fayetteville formations. *Mar. Pet. Geol.,* **31,** 43–52 (2012).
